# Immunosenescence: signaling pathways, diseases and therapeutic targets

**DOI:** 10.1038/s41392-025-02371-z

**Published:** 2025-08-06

**Authors:** Yichu Fu, Binhan Wang, Aqu Alu, Weiqi Hong, Hong Lei, Xuemei He, Huashan Shi, Ping Cheng, Xiangliang Yang

**Affiliations:** 1 College of Life Science and Technology, Huazhong University of Science and Technology, Wuhan, Hubei China; 2 Department of Biotherapy, Cancer Center, West China Hospital, Sichuan University, Chengdu, China; 3 Laboratory of Aging Research and Cancer Drug Target, State Key Laboratory of Biotherapy and Cancer Center, National Clinical Research Center for Geriatrics, West China Hospital, Sichuan University, Chengdu, China; 4 National Engineering Research Center for Nanomedicine, Huazhong University of Science and Technology, Wuhan, Hubei China

**Keywords:** Immunology, Diseases

## Abstract

Immunosenescence refers to the abnormal activation or dysfunction of the immune system as people age. Inflammaging is a typical pathological inflammatory state associated with immunosenescence and is characterized by excessive expression of proinflammatory cytokines in aged immune cells. Chronic inflammation contributes to a variety of age-related diseases, such as neurodegenerative disease, cancer, infectious disease, and autoimmune diseases. Although not fully understood, recent studies contribute greatly to uncovering the underlying mechanisms of immunosenescence at the molecular and cellular levels. Immunosenescence is associated with dysregulated signaling pathways (e.g., overactivation of the NF-κB signaling pathway and downregulation of the melatonin signaling pathway) and abnormal immune cell responses with functional alterations and phenotypic shifts. These advances remarkably promote the development of countermeasures against immunosenescence for the treatment of age-related diseases. Some anti-immunosenescence treatments have already shown promising results in clinical trials. In this review, we discuss the molecular and cellular mechanisms of immunosenescence and summarize the critical role of immunosenescence in the pathogenesis of age-related diseases. Potential interventions to mitigate immunosenescence, including reshaping immune organs, targeting different immune cells or signaling pathways, and nutritional and lifestyle interventions, are summarized. Some treatment strategies have already launched into clinical trials. This study aims to provide a systematic and comprehensive introduction to the basic and clinical research progress of immunosenescence, thus accelerating research on immunosenescence in related diseases and promoting the development of targeted therapy.

## Introduction

Immunosenescence, a concept proposed by Roy Walford in the 1960s, refers to the progressive remodeling and decline of immune function with aging. It is characterized by a diminished ability to respond to pathogens, reduced vaccine efficacy, and an increased risk of age-related diseases.^1^ This phenomenon encompasses both innate and adaptive immune dysregulation, which is characterized by thymic involution, chronic low-grade inflammation (“inflammaging”), and the accumulation of senescent cells (SnCs).^1,2^ Importantly, cellular senescence represents a stress response that induces irreversible cell cycle arrest, serving as a hallmark of aging.^3,4^ The pathological accumulation of SnCs not only amplifies age-related immunological alterations but also constitutes a key pathogenic driver of multiple age-associated comorbidities.^5,6^ Immune cells are pivotal modulators of cellular senescence.^5,7^ In this review, we focus on these immune populations, detailing how aging compromises their differentiation and functional capacity, while their dysregulation reciprocally accelerates the senescence process. Among these immune cell populations, T cells undoubtedly hold a predominant position. Thymic involution, impaired homeostatic proliferation of naïve T cells, and lifelong antigenic exposure collectively contribute to T cell senescence.^8^ This process drives the contraction of the naïve T cell compartment and expansion of the memory T cell pool, ultimately leading to a reduced diversity of the available T cell receptor (TCR) repertoire.^9^ Immunosenescence is also regulated by signaling pathways such as the nuclear factor-kappa B (NF-κB), mTOR, JAK-STAT, melatonin, and sirtuin pathways, whose dysregulation leads to aberrant immune responses and increased susceptibility to age-related diseases. Targeting these pathways may help mitigate immune decline in aging individuals.

Immunosenescence, characterized by dysregulation of signaling pathways and altered senescence/regulatory capacity across immune cell populations, may predispose individuals to diverse age-associated pathologies. These include neurodegenerative disorders, increased cancer incidence in geriatric populations, diminished efficacy of cancer immunotherapies, and heightened susceptibility to infectious and cardiovascular diseases. These diseases often present as multimorbidities, potentially leading to organ failure and death. Understanding the pathogenesis of these diseases and the driving factors behind their progression and deterioration can enhance our knowledge of immune system changes during immunosenescence. This insight can then guide the development of multifaceted strategies targeting immune organs, cells, and signaling pathways to restore immune competence, eliminate SnCs, and delay age-related dysfunction. Novel antiaging therapies targeting specific immune dysfunctions in elderly individuals are being proposed at an increasingly rapid pace, with some already entering clinical trials and demonstrating promising efficacy. This article systematically elaborates on various therapeutic interventions for immunosenescence.

In this review, we provide a systematic and comprehensive introduction to immunosenescence at three different levels: molecular, cellular and disease. More importantly, we provide a detailed summary of current strategies for targeting immunosenescence, ranging from targeted therapy to immunomodulation and lifestyle interventions. A concise summary of ongoing clinical trials targeting immune cells against immunosenescence is highlighted. By integrating these multifaceted strategies, this review not only addresses critical gaps in previous therapeutic frameworks but also highlights recent advancements and breakthroughs in fighting immunosenescence. The ultimate goal is to inspire future research to overcome existing research limitations and develop novel preventive and therapeutic approaches, thereby offering actionable solutions to mitigate aging-related diseases and extend the healthspan of the elderly population.

## Signaling pathways in immunosenescence

**Fig. 1:**
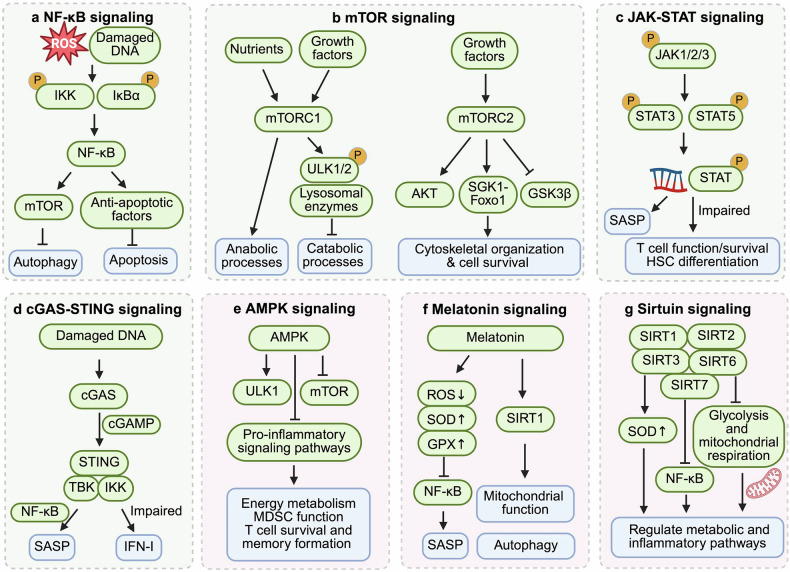
Signaling pathways associated with immunosenescence. Immunosenescence is associated with aberrant activation of various signaling pathways, such as upregulation of the NF-κB, mTOR, JAK-STAT, and cGAS-STING signaling pathways and downregulation of the AMPK, melatonin, and sirtuin pathways. **a** Accumulation of endogenous DNA damage and oxidative stress cause overactivation of NF-κB signaling, which transcriptionally activates the mechanistic target of mTOR and upregulates antiapoptotic proteins, thus impairing the induction of autophagy and apoptotic clearance of SnCs. **b** mTOR functions through two distinct complexes: mTOR complex 1 (mTORC1) and mTOR complex 2 (mTORC2). mTORC1 integrates signals from nutrients and growth factors to regulate various anabolic processes while inhibiting catabolic processes by phosphorylating ULK1/2 and sequestering lysosomal enzymes. mTORC2 regulates cytoskeletal organization and cell survival pathways through the activation of the AKT and SGK1-Foxo1 axes and the inhibition of GSK3β. **c** Overactivation of the JAK-STAT signaling pathway during aging contributes to immunosenescence by driving persistent inflammation and altering immune cell function and survival, including T cells and HSCs. **d** Accumulation of damaged DNA in aging cells activates the cGAS/STING pathway, which further induces NF-κB-dependent expression of inflammatory cytokines with impaired IFN-I production. **e** AMPK plays a crucial role in the regulation of cellular energy metabolism. It extends lifespan by promoting autophagy via mTOR inhibition and ULK1 activation. The activation of AMPK broadly suppresses proinflammatory signaling pathways, which inhibits the expansion and function of MDSCs and promotes the survival and memory formation of T cells. **f** Melatonin suppresses proinflammatory cytokines and enhances anti-inflammatory cytokines by inhibiting the NF-κB pathway. It directly scavenges ROS and upregulates the expression of antioxidant enzymes such as superoxide dismutase (SOD) and glutathione peroxidase (GPX), reducing oxidative damage and SASP accumulation in immune cells. Melatonin also mediates SIRT1 pathway activation, which optimizes mitochondrial function and autophagy. **g** Sirtuin family proteins play crucial roles in immune aging by regulating mitochondrial function, oxidative stress, and NF-κB signaling

Aberrant activation of various signaling pathways, including but not limited to the NF-κB, mTOR, JAK-STAT, cGAS (cyclic GMP-AMP synthase)-STING (stimulator of interferon genes), AMPK (AMP-activated protein kinase), melatonin, and sirtuin pathways, plays a crucial role in regulating immune function during aging. These pathways form a regulatory network to modulate immunosenescence (Fig. 1). Dysregulation of these pathways leads to impaired immune responses and increased susceptibility to age-related diseases. Understanding the intricate mechanisms by which these pathways influence immunosenescence is essential for developing targeted interventions to enhance immune function in elderly individuals.

### Upregulated signaling pathways

#### NF-κB signaling pathway

NF-κB is a transcription factor that can be activated during cellular damage and stress. The activity of NF-κB increases with aging and aging-related diseases due to the accumulation of endogenous DNA damage and oxidative stress. In aged mice, NF-κB activation has been observed in a variety of cell types.^10^ Genetic depletion or pharmacological inhibition of NF-κB decreases oxidative DNA damage and stress in aged mice, leading to delayed cellular senescence and age-related symptoms and pathologies.^10^ Persistent NF-κB activation drives inflammaging, which impairs immune surveillance, reduces T cell diversity, and promotes tissue degeneration.^11^ Reactive oxygen species (ROS) accumulation during aging can activate NF-κB via IκBα phosphorylation or IKK modulation at critical cysteine residues, impairing its DNA-binding capacity and disrupting redox homeostasis.^10,12,13^ Furthermore, NF-κB suppresses autophagy by transcriptionally activating the mechanistic target of mTOR, a key inhibitor of autophagic flux.^14^ Impaired autophagy in aged immune cells leads to the accumulation of damaged mitochondria and protein aggregates, exacerbating oxidative stress and inflammasome activation.^15^ Additionally, NF-κB enhances the survival of dysfunctional immune cells by upregulating antiapoptotic proteins, preventing the clearance of SnCs.^16^ This apoptotic resistance contributes to reduced immune diversity and increased cancer risk during aging. These findings underscore NF-κB as a central driver of immune aging, linking oxidative stress, inflammaging, autophagy suppression, and immune cell survival dysregulation.

#### mTOR signaling pathway

The mTOR signaling pathway serves as a central regulator of cell survival and growth and cell cycle progression.^17^ mTOR functions through two distinct complexes, mTOR complex 1 (mTORC1) and mTOR complex 2 (mTORC2), each of which play unique roles in cellular metabolism and immune function.^18^ mTORC1 integrates signals from nutrients and growth factors to regulate various anabolic processes, including protein synthesis, nucleotide production, lipid biosynthesis, and glycolysis, while inhibiting catabolic processes such as autophagy by phosphorylating ULK1/2 and sequestering lysosomal enzymes.^19–23^ The activation of mTORC1 has long been associated with cellular senescence^24^. However, in senescent CD8⁺ T cells, autophagy is not restored despite reduced mTORC1 activity, partly due to alternative inhibitory mechanisms such as chronic p38 MAPK activation, which impairs autophagy independently of mTORC1 and contributes to immune decline in senescent CD8⁺ T cells.^23,25^ Additionally, cytoplasmic p53 inhibits autophagy via mTOR activation under basal conditions, whereas nuclear p53 promotes autophagy through mTOR-independent mechanisms under stress.^26,27^ In addition to its role in autophagy regulation, mTORC1 also plays an important role in T cell function. In aged mice, in vitro inhibition of mTORC1 with metformin or everolimus increased IL-2 production and T cell proliferation and reduced oxidative stress in CD4⁺ T cells^28^. Moreover, in elderly humans, low-dose mTORC1 inhibition with RAD001 and BEZ235 reduced infection rates over 12 months, indicating an enhancement of immune function.^29^

mTORC2 has been increasingly recognized for its role in immune aging. In aged murine CD4⁺ T cells, increased mTORC2 signaling is associated with impaired TCR responsiveness and reduced proliferative capacity. These functional defects are related to mTORC2-mediated dysregulation of cytoskeletal organization (including actin polymerization) and cell survival pathways.^30^ In vivo studies in lymphocytic choriomeningitis virus-infected mice have shown that mTORC2 prevents ferroptosis in virus-specific memory CD4⁺ T cells by limiting lipid peroxidation and mitochondrial ROS accumulation, primarily through activation of AKT and inhibition of GSK3β, thereby supporting their long-term survival.^31^ Moreover, mTORC2 regulates CD8⁺ T cell differentiation via the SGK1-Foxo1 axis. The inhibition or genetic deletion of SGK1, a downstream effector of mTORC2, promotes the formation of memory precursors of CD8⁺ T cells and enhances their long-term survival.^32^

#### JAK-STAT signaling pathway

The JAK-STAT signaling pathway is fundamental to immune regulation and plays key roles in infection defense, immune tolerance, and tumor surveillance. During aging, the JAK-STAT signaling pathway is dysregulated, contributing to immunosenescence by driving persistent inflammation and altering immune cell function. JAK-STAT pathway alterations impair immune homeostasis by affecting immune cell development and function. Hyperactivation of STAT3 enhances the production of proinflammatory cytokines, including interleukin (IL)-6 and IL-23, promoting the senescence-associated secretory phenotype (SASP) and sustaining inflammatory signaling.^33^ JAK1/2 activation further amplifies the SASP, accelerating immune aging. Additionally, JAK3 and STAT5B mutations impair Foxp3 expression, disrupting regulatory T cell (Treg)-mediated immune tolerance.^34^ JAK3 mutations result in defective T and natural killer (NK) cell maturation, weakening immune responses against infections and tumors. Overactivation of STAT3 skews the immune balance by promoting T helper 17 (Th17) cell expansion while suppressing Treg function. Moreover, excessive JAK-STAT activation disrupts hematopoietic stem cell (HSC) differentiation, favoring myeloid rather than lymphoid lineage commitment, a hallmark of immune system aging.^35^

#### cGAS-STING

The cGAS-STING pathway senses cytosolic DNA under certain conditions, such as DNA damage, mitochondrial dysfunction, or nuclear envelope disruption. The accumulation of damaged DNA in the cytoplasm, which serves as a damage-associated molecular pattern (DAMP) to be recognized by DNA sensors, including the cGAS-STING pathway, is observed in aging cells.^36^ Through the cGAMP–STING–TBK/IKK axis, it induces NF-κB-dependent expression of inflammatory cytokines such as IL-6 and CXCL10, promoting the SASP.^37^ Notably, aging immune cells exhibit impaired secretion of type I interferons (IFN-I), which are downstream effector molecules of cGAS-STING signaling. For example, aging plasmacytoid dendritic cells (pDCs) exhibit limited production of IFN-I due to impaired IRF7 phosphorylation, resulting in decreased presentation of antigens to T lymphocytes.^38^

### Downregulated signaling pathways

#### AMPK signaling pathway

AMPK is a pivotal serine/threonine protein kinase that plays an extensive role in the regulation of cellular energy metabolism^39^. It is critically involved in maintaining cellular homeostasis, mitigating oxidative stress, promoting cell survival and growth, and modulating cell death and autophagy^40^. As a central energy sensor, AMPK extends lifespan by promoting autophagy via mTOR inhibition and ULK1 activation, enhancing mitochondrial function through PGC-1α/SIRT1 signaling and improving the NAD^+^/NADH balance, suppressing inflammatory responses via NF-κB inhibition, and modulating stress resistance via the FOXO3/p53 pathways, thereby linking metabolic regulation to aging suppression^41^. The activation of AMPK broadly suppresses proinflammatory signaling pathways, including the JAK-STAT, NF-κB, C/EBPβ, CHOP, and HIF-1α pathways, which in turn inhibits the expansion and immunosuppressive function of myeloid-derived suppressor cells (MDSCs)^42^. Moreover, AMPKα1 is essential for CD8⁺ T cell memory formation because it senses glucose deprivation and suppresses mTORC1 activity, as AMPKα1-deficient CD8⁺ T cells fail to survive metabolic stress during immune contraction and exhibit impaired secondary responses^43^. In highly differentiated human CD4⁺ T cells (the CD27⁻CD28⁻ subset), AMPK activation under metabolic stress or DNA damage recruits TAB1 to induce p38 autophosphorylation, leading to telomerase suppression and proliferative arrest^44^. Given its regulatory effects on immune signaling and inflammation, AMPK may play a role in modulating immunosenescence, although further studies are needed to elucidate this potential connection.

#### Melatonin signaling pathway

Melatonin, a hormone produced by the pineal gland that plays a pivotal role in regulating circadian rhythms, significantly decreases in level as age progresses, manifesting as a deterioration of circadian rhythmicity.^45^ Melatonin counteracts immunosenescence via a multitarget regulatory network spanning cytokine balance, oxidative stress defense, immune cell functional restoration, signaling pathway crosstalk, disease-specific interventions, and circadian rhythm integration. Specifically, melatonin suppresses proinflammatory cytokines (e.g., IL-1β, IL-6, tumor necrosis factor-α (TNF-α), and interferon-γ (IFN-γ)) and enhances anti-inflammatory cytokines (e.g., IL-4 and IL-10) by inhibiting the NF-κB pathway, although its low-dose transient proinflammatory effects highlight dose- and pathology-dependent dynamics.^46,47^ It directly scavenges ROS and upregulates the expression of antioxidant enzymes such as superoxide dismutase (SOD) and glutathione peroxidase (GPX), reducing oxidative damage and SASP accumulation in immune cells.^46–48^ Through an “antioxidant cascade”, melatonin metabolites such as N1-acetyl-N2-formyl-5-methoxykynuramine (AFMK) and N1-acetyl-5-methoxykynuramine (AMK) further neutralize free radicals, protecting mitochondrial integrity, proteins, and DNA from oxidative destruction. At the immune cell level, melatonin enhances CD4^+^/CD8^+^ T cell proliferation and antigen responsiveness, modulates the Treg/Th1/Th2 balance, promotes macrophage polarization toward the anti-inflammatory M2 phenotype, and augments NK cell cytotoxicity.^46,47^ These effects are mediated by SIRT1 pathway activation, which optimizes mitochondrial function and autophagy, and by miRNA-dependent regulation (e.g., miR-146a targeting Nrf2/NF-κB pathways), although the mechanisms vary across cell types and microenvironments.^46,47^ Epigenetically, melatonin may inhibit histone deacetylases (HDACs) and modulate miRNA expression to reverse age-associated proinflammatory gene silencing in senescent immune cells, restoring functional competence.^49^ As a core circadian regulator, melatonin stabilizes clock genes (e.g., CLOCK protein), reduces cortisol-mediated immunosuppression, and coordinates rhythmic immune cell activities (e.g., diurnal fluctuations in macrophage phagocytosis), systemically delaying immunosenescence.^46,49^

#### Sirtuin signaling pathway

Sirtuin family proteins play crucial roles in immune aging by regulating mitochondrial function, oxidative stress, and NF-κB signaling, thereby maintaining immune homeostasis across various immune cell types. In HSCs, sirtuin 3 (SIRT3) preserves genomic stability and mitochondrial integrity, delaying cellular aging by increasing superoxide dismutase 2 (SOD2) activity and reducing oxidative stress.^50^ Restoring NAD^+^ levels can further reactivate SIRT3, improving stem cell reprogramming and lifespan extension.^51^ Within the innate immune system, SIRT1 and SIRT6 mitigate inflammatory responses in macrophages by inhibiting NF-κB signaling and suppressing excessive TNF-α and IL-1β expression, promoting endotoxin tolerance.^52^ In dendritic cells (DCs), SIRT1 modulates autophagy and cytokine secretion, enhancing antiviral responses while preventing excessive Th2/Th17-mediated inflammatory reactions.^53^ In NK cells, SIRT2 and SIRT6 contribute to exhaustion in colorectal cancer, suppressing NK cytotoxicity by downregulating glycolysis and mitochondrial respiration. Silencing these proteins restores the antitumor function of NK cells.^54,55^ Within adaptive immunity, SIRT1 is essential for T cell activation and peripheral tolerance, preventing autoimmune diseases by suppressing AP-1 transcription and IL-2 production in the absence of CD28 costimulation.^56^ Furthermore, both SIRT1 and SIRT7 regulate B cell class-switch recombination (CSR), influencing immunoglobulin (Ig) maturation.^57^ Overall, the sirtuin family acts as a critical regulator of immune aging by fine-tuning metabolic and inflammatory pathways in multiple immune cell types. Targeting these proteins may provide novel therapeutic strategies to mitigate immune decline and promote healthier aging.

## Cellular mechanisms of immunosenescence

**Fig. 2:**
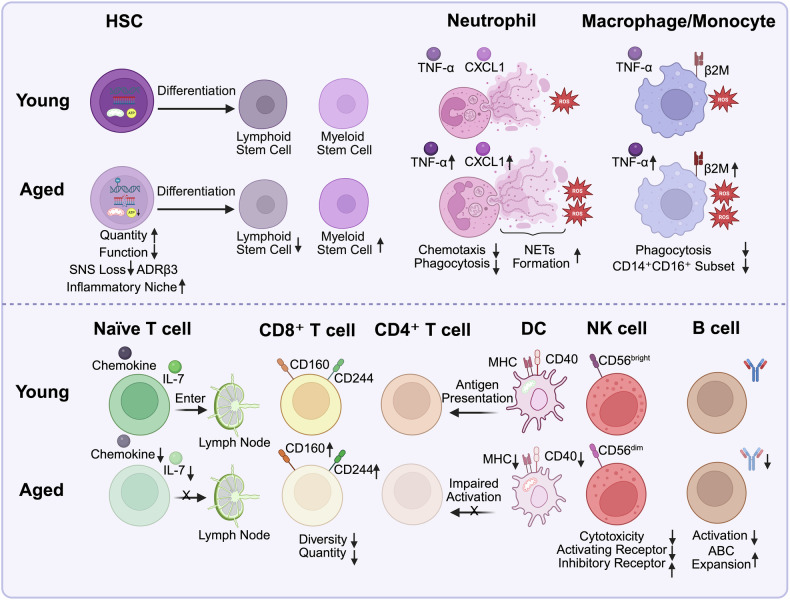
Cellular mechanisms of immunosenescence. Aging-induced alterations in various immune cell populations are depicted with young cells in the top row and aged cells in the bottom row. (1) HSC: Aging increases the number of HSCs but weakens their function. SNS degeneration decreases ADRβ3 signaling, generating an inflammatory niche. Myeloid-biased differentiation reduces lymphoid output, weakening adaptive immunity. (2) Neutrophils: Aged neutrophils exhibit prolonged lifespan, hypersegmentation, and impaired chemotaxis but enhanced CXCL1-driven recruitment. Elevated NET formation, ROS, and TNF-α production promote chronic inflammation. (3) Macrophages and Monocytes: Aging increases the proportion of CD14⁺CD16⁺ monocytes, which exhibit a proinflammatory phenotype with increased TNF-α and IL-6 production. Reduced phagocytosis causes debris accumulation and chronic inflammation. Elevated ROS, NO, and β2M contribute to metabolic diseases and cognitive decline. (4) T cells: Aging reduces the levels of IL-7 and chemokines, impairing naïve T cell survival, proliferation, and lymph node entry and limiting renewal. Aging decreases CD8⁺ T cell diversity and number. CD160 and CD244 expression increases, resembling an exhausted phenotype. Aged CD8⁺ T cells show reduced cytotoxicity and produce less IFN-γ, granzyme B, and perforin. CD4⁺ T cell activation decreases in part due to elevated PD-1 expression. (5) Aged DCs have weaker antigen presentation (MHC/CD40 downregulation), resulting in weaker CD4⁺ T cell responses. (6) NK cells: Aging reduces the number of CD56^bright^ NK cells and their activating receptors while increasing the number of inhibitory receptors (KIRs), impairing cytotoxicity. Degranulation and perforin secretion decline. NK cells shift toward a CD56^dim^ subset, where they secrete more proinflammatory cytokines, contributing to chronic inflammation. (7) B cells: In elderly individuals, antibody production and class switching decline due to CD40 downregulation and weakened BCR signaling. ABC expansion disrupts immune balance, weakening humoral immunity

The aberrant signaling pathways during aging result in the dysfunction of immune cells, which interferes with almost all kinds of immune cells, ranging from HSCs to mature immune cells. Senescent immune cells under excessive loading contribute to age-related pathological changes, which can progress to age-related diseases. The proportion of HSCs, the progenitor cells of immune cells, is significantly increased in elderly individuals. Despite an increased tendency for self-renewal, their overall regenerative capacity declines due to impaired differentiation potential and functional deterioration. This is characterized by skewed hematopoietic output, accumulation of replication stress, and reduced adaptability to transplantation or hematopoietic challenges.^58,59^ Aged HSCs exhibit a myeloid-biased differentiation tendency, leading to a reduced generation of lymphoid lineage cells (T and B cells) and a decline in adaptive immune function (Fig. 2).^60,61^ Moreover, aging HSCs displayed impaired function, including reduced blood production and impaired engraftment after transplantation. Replication stress is a key driver of functional decline in aged HSCs, which is due to reduced expression of mini-chromosome maintenance genes and impaired DNA replication dynamics.^59^

HSC aging can be induced by various mechanisms. Markus’s team reported that the myeloid-biased output of HSCs was mediated by interleukin-1 (IL-1) signaling in a mouse model.^62^ Knocking out IL-1 receptor 1 (IL-1R1) or pharmacologic inhibition of IL-1 signaling in older mice reversed the myeloid-biased hematopoietic output. Degeneration of the sympathetic nervous system (SNS) in the bone marrow microenvironment is another contributor to HSC aging. Interfering with the SNS nerves of adrenoreceptor β3 (ADRβ3) signaling resulted in premature HSC aging in young mice, whereas stimulating ADRβ3 in older mice rejuvenated the functions of HSCs.^63^ Zinc finger proteins in aged murine HSCs contribute to increased platelet bias and sustained myeloid HSC bias while suppressing lymphoid lineage output.^64^ Moreover, epigenetic changes in aged HSCs impair differentiation, including disrupted DNA methylation and histone modifications.^58^ The decrease in autophagy in aged HSCs leads to the accumulation of active and healthy mitochondria and increased metabolism, particularly increased oxidative phosphorylation, promoting accelerated myeloid differentiation of HSCs.^65^

### Immunosenescence and aging-related dysregulation of myeloid lineage cells

Myeloid lineage cells are composed of various innate immune cells, such as neutrophils, macrophages, and monocytes. The myeloid-biased differentiation of aged HSCs leads to increased production of myeloid immune cells. In addition, mature myeloid immune cells display altered phenotypes and functions with elevated production of proinflammatory cytokines such as TNF-α and IL-6, which are closely related to age-related senescent inflammation, termed senoinflammation.^66–69^ Normally, neutrophils are short-lived innate immune cells. During aging, neutrophils display an extended lifespan and abnormal phenotypic features such as hypersegmentation in secondary lymphoid organs and bone marrow.^70,71^ Under inflammatory conditions, aging neutrophils can exhibit alterations in their functional state with increased integrin activation and the formation of neutrophil extracellular traps (NETs).^72^ Aged neutrophils remain active under lipopolysaccharide (LPS) stimulation, releasing many cytokines.^73^ Dysregulated activation of aging neutrophils may be associated with compromised calcium signaling pathways and increased metabolic byproducts (e.g., spontaneous ROS production and increased NAD^+^ levels).^74,75^ Notably, aging can also cause dysregulation of neutrophil recruitment in response to aberrant chemokine signaling and inflammatory responses. During influenza infection in mice, increased neutrophil recruitment to aging lungs or livers was observed upon C-X-C motif chemokine ligand 1 (CXCL1) stimulation, resulting in devastating inflammation and increased mortality.^76,77^ The upregulation of junctional adhesion molecule-C also promotes the accumulation of neutrophils in the lungs, leading to acute lung injury.^78^ In contrast, neutrophil depletion limits the secretion of neutrophil-activating cytokines and reduces mortality and long-term functional benefits in an ischemic stroke model in aged mice.^79^

Aging alters the phenotype and function of monocytes, increasing the proportions of nonclassical monocytes and intermediate monocytes.^67–69^ The proportion of the CD14^+^CD16^+^ subset of monocytes was elevated with downregulated expression of CX3CR1 and HLA-DRA during aging, whereas the CD14^+^CD16^-^ subset was decreased.^69,80^ During aging, the level of β2-microglobulin (a component of major histocompatibility complex (MHC)-I) in plasma increases, leading to a proinflammatory phenotype in monocytes, cognitive decline, and regenerative impairments in the adult brain.^81,82^ Aging also promotes the proinflammatory polarization of monocytes by increasing the plasma saturated fatty acid concentration, thereby increasing the production of IL-6 and TNF-α but inhibiting the production of IL-10 and transforming growth factor-β (TGF-β1).^83^ Overproduction of IL-6 and TNF-α in the peripheral blood of aging monocytes may be related to human Toll-like receptor 2/6 (TLR2/6) signaling instead of TLR1/2 signaling.^84^ Epigenetic alterations such as histone modifications (e.g., H3K9me3 loss) contribute to the upregulation of inflammatory genes and an imbalance in macrophage polarization, further reinforcing systemic inflammation.^85^ Moreover, the phagocytic activity of macrophages and monocytes significantly decreases during aging, leading to the accumulation of unphagocytosed debris, chronic sterile inflammation, and the exacerbation of tissue aging and damage.^86^

### Immunosenescence and aging-related dysregulation of lymphoid lineage cells

Lymphoid cells are composed of innate (such as NK cells) and adaptive immune cells (such as T cells and B cells). T and B cells can be further divided into a number of different subtypes. In general, aging is related to reduced production and impaired function of adaptive immune cells but elevated memory cell expansion, including both T and B cells.^87–91^ Thymic involution is a core characteristic of immunosenescence and is characterized by gradual shrinkage of the thymus and a significant reduction in thymic epithelial tissue, leading to a pronounced decline in the production of T cells, particularly naïve T cells.^87–89^ As the thymus shrinks, TCR diversity also decreases, impairing the ability of the immune system to respond effectively to novel pathogens^92^. In naïve T cells, aging disrupts their ability to enter and interact with survival factors, impairing their proliferation and function.^93^ Animal studies have indicated that age-related disruption of naïve T cell survival and homeostasis depends on alterations in the secondary lymphoid environment and IL-7 (a maintenance factor) signaling.^94,95^ Defects in the function of stromal cells in the secondary lymphoid organs of aged individuals play crucial roles in immune cell migration, activation, and survival of naïve T cells.^96^

A functional decline was observed in aging T and B cells. Unlike anergy and exhaustion, T cell senescence is an irreversible process.^97,98^ Senescent T cells are characterized by a phenotypic shift with the downregulation of the costimulatory molecules CD27 and CD28 and the upregulation of the killer cell lectin-like receptor subfamily G and CD57.^99^ Moreover, aging induces the expression of immune checkpoint-related molecules such as lymphocyte-activation gene 3 (LAG-3), programmed death protein 1 (PD-1), and cytotoxic T-lymphocyte-associated protein 4 (CTLA-4).^100,101^ This is coupled with the upregulation of the cell cycle regulators P16, P21, and P53, leading to cell cycle arrest and diminished proliferative capacity.^102^ Compared with CD44^low^CD8^+^ T cells, CD8^+^ T cells in aging individuals predominantly express high levels of CD44, which are of low quality.^101^ CD44^high^CD8^+^ T cells in aged mice presented similar transcriptional properties to exhausted CD8^+^ T cells during chronic viral infection and highly expressed inhibitory molecules, including CD160, CD244, LAG-3, and PD-1.^101^ Additionally, senescent T cells show reduced cytotoxicity with decreased production of functional immune molecules, including IFN-γ, granzyme B, and perforin.^98,100,103^ Compared with those of CD8^+^ T cells, the diversity and output of CD4^+^ T cells are more stable, although some studies have indicated the accumulation of CD4^+^ T cells expressing PD-1 and Tregs (CD4^+^CD25^+^Foxp3^+^) during aging.^104–107^ Notably, in Tregs, DNA methylation at the Foxp3 locus plays a crucial role in maintaining their anti-inflammatory function. Age-related alterations in this methylation pattern impair Treg activity, leading to dysregulated immune tolerance and contributing to the proinflammatory milieu associated with aging.^108^ With respect to B cells, aging B cells exhibit defects in antibody production and immunoglobulin (Ig) class switching.^90,91^ This is associated with reduced activation of the transcription factor 3/E47 transcription factor in aged B cells.^91^ Aging also decreases the AID enzyme (activation-induced cytidine deaminase), which is responsible for class-switch recombination and the production of high-affinity antibodies.^90^ Moreover, aging B cell activation was impaired by the downregulation of CD40 expression, which reduced responsiveness to B cell receptor (BCR) stimulation.^109^ During viral infection, aging is associated with a decline in B cell frequencies, reduced antibody responses, and weaker protection, ultimately reducing vaccine efficacy in elderly individuals.^110^

Some studies have indicated that elderly individuals possess a greater number and proportion of memory T cells (especially memory CD8^+^ T cells) and memory B cells.^111,112^ Chronic antigen stimulation and age-related inflammation contribute to this shift, accelerating naïve T cell decline while promoting memory T cell dominance.^113^ Vesna et al. discovered novel human memory CD8^+^ T cells with a naïve phenotype that accumulate with aging and can rapidly respond to persistent antigens by producing various cytokines.^112^ Age-related accumulation of memory-phenotype CD8^+^ T cells partially compensates for the loss of naïve T cells, enhancing responses to previously encountered pathogens.^114,115^ Age-associated B cells (ABCs), most of which are antigen-experienced memory B cells, are induced upon exposure to microbial infection and play a crucial role in pathogen clearance and control.^116,117^ TLR signaling is essential for B cell activation and differentiation, particularly for IgM⁺ memory B cells. In vitro studies have shown that TLR7 and TLR9 stimulation can expand IgM⁺ memory and plasma cells, promoting IgM secretion.^118^

NK cells are widely distributed cytotoxic innate lymphoid cells that can rapidly recognize and kill cancer cells or pathogen-infected cells.^119^ Aging impairs NK cell-mediated immune responses by reducing the number of mature NK cells and altering their activity. The number and proportion of NK cells gradually increased with age, but the CD56^bright^ mature cell population decreased.^120,121^ Moreover, aging inhibited the expression of activating receptors but promoted the upregulation of killer cell inhibitory receptors (KIRs) on CD56^bright^ NK cells.^122,123^ These changes weaken NK cell cytotoxicity to eliminate tumor cells and virus-infected cells.^124^ Aged NK cells exhibit reduced degranulation and impaired perforin secretion, which may be linked to alterations in Ca²⁺-dependent exocytosis regulated by Munc13-4, a key protein in NK cell cytotoxic granule release.^125^ At the molecular level, aging leads to the downregulation of key transcription factors such as EOMES and T-bet, hindering NK cell maturation.^126,127^ Epigenetic changes, such as reduced miR-181a-5p expression, contribute to an immature NK cell phenotype and functional defects.^128^ Additionally, nonhematopoietic cells, such as bone marrow stromal cells, may disrupt NK cell function through unknown signals.^129^ Aging also causes upregulation of the CD56^dim^ immature NK subset with increased secretion of proinflammatory cytokines, exacerbating chronic inflammation.^121^ In conclusion, NK cell aging is characterized by hindered maturation, functional impairment, and a shift toward a proinflammatory phenotype.

DCs are a type of cell of special origin that can be derived from both lymphoid stem cells and myeloid stem cells. DCs play a vital role in activating adaptive immunity as professional antigen-presenting cells. In elderly individuals, the capacity of DCs to phagocytose antigens and migrate is significantly impaired.^130^ Moreover, DCs in aged mice presented downregulation of MHC and CD40 expression, weakening their ability to release proinflammatory cytokines in response to LPS stimulation and inducing CD4^+^ T cell immunity.^131,132^ Aged DCs exhibit dysfunctional mitochondria, with impaired energy production and increased oxidative stress^133^. Restoring mitochondrial health could increase the antigen-presenting ability of aged DCs.^133^ Aging-associated upregulation of WNT5A in the hematopoietic system activates the noncanonical WNT/CDC42 pathway, leading to impaired differentiation of plasmacytoid and conventional dendritic cells. Pharmacological inhibition of this pathway may restore DC development and function in aging.^134^ In aged mice, bone marrow-derived DCs exhibit increased IL-23 production and increased p19 mRNA expression upon TLR activation. This upregulation is associated with chromatin remodeling, which is characterized by di- and tri-methylation of histone H3K4 and preferential binding of c-Rel at the p19 promoter, contributing to age-related inflammatory responses.^135^ The increased basal activation levels of aged DCs disrupt respiratory epithelial function by altering cytokine and chemokine secretion, contributing to chronic airway inflammation and heightened susceptibility to respiratory infections in elderly individuals.^136^ In autoimmune diseases, DCs from aged mice exhibit reduced expression of TRIM28, a nuclear protein that silences gene expression. This resulted in increased T cell differentiation toward inflammatory effector cells.^137^ Therefore, aging has a significant effect on the activation, migration, and functions of DCs.

### Effects of microbiome & sex differences on immunosenescence

During the aging process, the composition of the gut microbiota undergoes alterations and is closely associated with the progression of immunosenescence. In aged mice, the levels of anti-inflammatory bacteria such as *Faecalibacterium prausnitzii* and *Bifidobacteria spp*. are reduced. In aged individuals, the gut microbiota shifts toward a more *Bacteroidetes*-dominant structure, along with a decreased *Firmicutes/Bacteroidetes* (F/B) ratio.^138,139^ The F/B ratio is critical for the production of short-chain fatty acids (SCFAs), which play essential roles in maintaining intestinal and immune homeostasis. SCFAs, such as butyrate, inhibit HDAC activity and enhance Treg function.^140,141^ Age-related dysbiosis leads to increased intestinal permeability, allowing proinflammatory microbial products such as LPS to enter the circulation. This results in the upregulation of inflammatory molecules such as IL-6 and TNF-α, which promote chronic inflammation and further enhance the SASP.^142^ Notably, the gut microbiota of healthy centenarians is enriched with anti-inflammatory taxa such as *Akkermansia* and *Christensenellaceae*, which are potential producers of SCFAs.^143^ In addition, *Lactobacillus* is more abundant in healthy centenarians and produces the antioxidant L-ascorbic acid, which helps scavenge free radicals and reduce oxidative stress.^144^ Furthermore, *Bifidobacterium longum subsp. longum* has been shown to modulate the host immune transcriptome and suppress proinflammatory cytokine expression.^145^ Therefore, maintaining gut microbial homeostasis and enhancing both anti-inflammatory and antioxidant capacity may represent promising strategies for delaying immune aging and promoting healthy longevity.

Sex differences in immune aging are multifaceted, encompassing immune cell composition, functional responses, genetic background, and hormonal regulation. Females tend to have (1) higher CD4^+^ T cell levels and CD4/CD8 ratios, (2) a more activated phenotype in circulating monocytes, (3) higher frequencies of B cells, and (4) greater interferon production from pDCs. In contrast, males accumulate more senescent CD8^+^ T cells, reflecting a sex-biased trajectory of immunosenescence.^146–148^ These sex differences are concurrently influenced by sex hormones, with estrogens enhancing multiple immune parameters in a dose-dependent manner. Low concentrations of estrogens promote Th1 proinflammatory responses, whereas higher levels induce Th2 humoral immunity.^149^ Moreover, estrogens confer antioxidant advantages by inhibiting ROS-producing enzymes and upregulating antioxidant systems such as SOD and GPX in both rodents and humans.^149^ Sex chromosomes themselves also shape immune aging. At the molecular level, X-linked immune genes such as *Tlr7* escape inactivation in females, promoting stronger antiviral responses, whereas age-related mosaic loss of the Y chromosome in males impairs leukocyte gene regulation.^147^ Functionally, females produce stronger vaccine-induced antibody responses than males do, underscoring the need for sex-specific immunization strategies for elderly individuals.^150^ As global aging accelerates, elucidating sex-based immune differences is critical for designing targeted interventions for age-related diseases.

## Immunosenescence-related diseases

### Neurodegenerative diseases

**Fig. 3:**
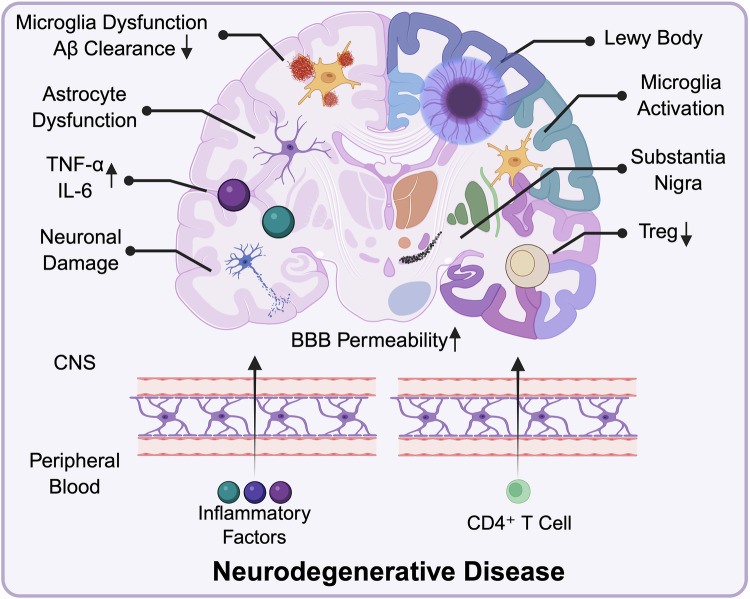
Immunosenescence in neurodegenerative diseases. In AD, immunosenescence and inflammaging drive chronic neuroinflammation, fostering neuronal damage and impairing Aβ clearance via dysfunctional microglia. Aβ deposition triggers the uncontrolled activation of microglia and astrocytes. Increased BBB permeability allows Th1/Th17 infiltration and the secretion of proinflammatory cytokines, exacerbating neurodegeneration, whereas Tregs help suppress inflammation and clear Aβ. In PD, misfolded α-synuclein aggregates into Lewy bodies, causing dopaminergic neuron loss in the substantia nigra. Peripheral CD4⁺ T cell infiltration and IL-17 signaling drive neuroinflammation and neuronal apoptosis. Activated microglia amplify this process by fostering a proinflammatory environment, whereas reduced Tregs fail to suppress excessive immune activation

Aging leads to the establishment of an interdependent relationship between the nervous and immune systems, where changes in one system influence the other (Fig. 3).^151^ In elderly individuals, inflammaging, along with peripheral immunosenescence, modulates the activity and reactivity of neuronal immune cells. This results in chronic, low-grade inflammation within the central nervous system (CNS), referred to as neuro-inflammaging,^151^ with biomarkers including C-reactive protein (CRP), IL-6, and TNF-α.^152^ Preclinical studies have suggested that cytokine-driven glial activation may contribute to memory impairment and cognitive decline.^152,153^ These cytokines can enter the nervous system from the periphery, with the largest source being autoreactive T cells derived from the atrophied thymus, which strongly contribute to neurodegeneration.^106^ Immunosenescence and inflammaging together accelerate brain aging, cognitive decline, and memory loss. This interplay between the immune system and nervous system is evident in neurodegenerative diseases such as Alzheimer’s disease (AD) and Parkinson’s disease (PD), fuelling dementia progression.^154^

AD is a progressive neurodegenerative disorder that is difficult to detect in its early stages, with cognitive impairment and memory problems manifesting in later stages and worsening over time.^155^ It is characterized by abnormal extracellular amyloid-beta (Aβ) aggregates, which form diffuse and neuritic plaques, as well as hyperphosphorylated tau aggregates, which form intraneuronal neurofibrillary tangles.^156^ With age, the failure of this immune barrier makes it more difficult for the innate immune system to respond. Senescent microglia (resident macrophages in the brain) exhibit increased proliferation and proinflammatory cytokine production but a reduced ability to clear Aβ, contributing to its accumulation in the brain.^157^ Conversely, the deposition of Aβ triggers the uncontrolled activation of microglia and astrocytes, which are ostensibly responsible for the clearance of damaged cells. However, this aberrant activation state precipitates excessive inflammation.^158,159^ Sustained neuroinflammation fosters mitochondrial dysfunction, neuronal injury, and cell death, which may contribute to the cognitive decline observed in neurodegenerative disorders, particularly memory impairment and, in some cases, language deficits.^160,161^ Furthermore, Aβ can function as a DAMP and activate the inflammasome via the TLR pathway, leading to the production of inflammatory cytokines such as IL-1β. This mechanism also significantly contributes to the pathogenesis of AD.^162^ Additionally, the meningeal lymphatic system is crucial for Aβ clearance, and its function is impaired with age, leading to cognitive decline. Enhancing meningeal lymphatic drainage can promote Aβ clearance and improve cognitive function in a mouse model.^163^ Changes in T cell senescence are also associated with cognitive decline.^164^ Compared with healthy young or elderly individuals, patients with AD exhibit an expansion of senescent T cells (within both the CD4^+^ and CD8^+^ populations) in the peripheral blood.^165^ CD4^+^ effector T cells in peripheral blood can differentiate into Th17 and Th1 subsets. Th1 and Th17 cells secrete cytokines that disrupt the tight junctions of the blood‒brain barrier (BBB), allowing inflammatory factors such as TNFα, IL-1β, and IL-6 to enter the brain, thereby accelerating the progression of AD.^166,167^ Tregs play crucial roles in suppressing neuroinflammation and facilitating the clearance of amyloid plaques. Their anti-inflammatory function not only helps regulate the immune response but also promotes cognitive function. Depletion of Tregs has been shown to improve cognitive performance and enhance the clearance of amyloid plaques in mouse models, highlighting their significant role in AD pathology.^168,169^ Aging affects B cell function, reducing antibody specificity and memory B cell formation, increasing the susceptibility of elderly individuals to infections and inflammation.^170,171^ In AD patients, B cells may produce autoantibodies that recognize misfolded Aβ peptides, potentially assisting microglia in clearing plaques.^172^ However, single-cell analyses revealed that microglia undergo dynamic transcriptional reprogramming into disease-associated microglia with altered phagocytic capacity.^173^ These observations suggest a possible interplay between B cell-mediated humoral immunity and microglial phagocytic function in AD pathogenesis.

PD, a neurodegenerative disorder closely linked to aging, involves a complex interplay between immunosenescence and neuroinflammation. Pathologically, PD is characterized by the misfolding of α-synuclein, leading to Lewy body formation and dopaminergic neuron loss in the substantia nigra.^174,175^ Oxidative stress, proteasome dysfunction, and protein aggregation, changes that frequently occur during aging, have been implicated in the pathogenesis of PD.^176^ While it remains debated whether neuroinflammation initiates or results from neurodegeneration, systemic inflammation is known to amplify CNS pathology. Activated microglia within the brain exacerbate neuronal damage by promoting a proinflammatory environment, further driving neuroinflammation and neurodegeneration in PD.^177^ Peripheral inflammation is associated with the activation of immune cells, including T cells, macrophages, and monocytes, which can breach the BBB due to their increased permeability in this disease state.^178^ This allows peripheral immune cells to infiltrate the central nervous system, contributing to neuroinflammation and accelerating neuronal damage.^179^ In PD, changes in immune cell function, particularly the accumulation of senescent T cells, significantly exacerbate the progression of the disease.^180^ Peripheral CD4^+^ T cells infiltrating the brain not only serve as primary mediators of dopamine toxicity but also respond to α-synuclein, promoting neuronal cell death. Additionally, they can influence oxidative stress and mitochondrial dysfunction, ultimately contributing to neurodegeneration.^181^ Like those in AD patients, the brain tissues of PD patients also contain Th17 cells. The IL-17 secreted by these cells binds to IL-17 receptors (IL-17Rs) expressed in midbrain neurons, inducing neuronal death through the upregulation of NF-κB and downstream signaling pathways, as demonstrated in human iPSC-derived neuronal models.^182^

### Cancer

**Fig. 4:**
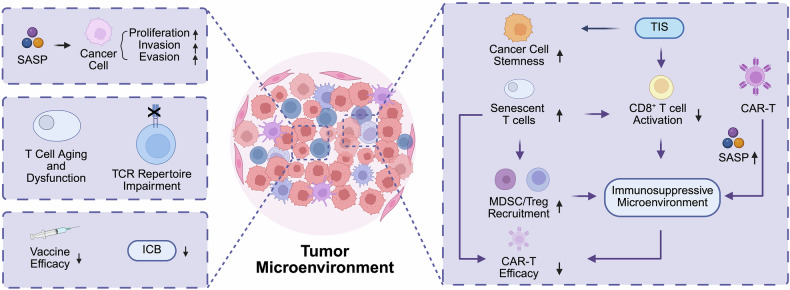
Immunosenescence in cancer. In the left part of the figure, the SASP enhances tumor growth, invasion, and immune evasion, exacerbating immune suppression. Aging-related T cell exhaustion and an impaired TCR repertoire weaken immune surveillance. Additionally, reduced vaccine efficacy and diminished immune checkpoint blockade (ICB) responses are observed in the senescent TME. In the right part of the figure, therapy-induced senescence (TIS) reprograms tumor cells toward stem-like phenotypes and suppresses CD8⁺ T cell activation. This suppression of CD8⁺ T cell activity subsequently contributes to the development of an immunosuppressive environment. A similar effect is observed with the accumulation of senescent T cells, which also suppress CD8⁺ T cell activation and thereby promote immune suppression. Moreover, senescent T cells can recruit MDSCs and Tregs and further induce senescence in neighboring effector T cells, thereby reinforcing immune suppression and impeding effective antitumor responses within the immunosuppressive milieu. Furthermore, CAR-T cell immunotherapy itself can induce SASP-related cytokines. This adverse environment, together with the presence of senescent T cells, synergistically undermines the efficacy of CAR-T cell therapy

As individuals age, the risk of cancer increases, partly due to immune senescence, which involves a decline in immune system function and contributes to tumorigenesis (Fig. 4). In the tumor microenvironment, SnCs secrete SASP components.^183^ The SASP plays a crucial role in mediating the crosstalk between SnCs and their neighboring cells, often exacerbating pathways of cellular damage and leading to the disruption of immune balance.^184–186^ The role of the SASP in tumors can be summarized as follows: (1) It promotes the growth and proliferation of tumor cells. For example, fibroblast growth factor 10 (FGF10) secreted by senescent mesenchymal cells induces multifocal prostate cancer,^187^ and the expression of fibroblast growth factor 19 by skeletal muscle cells can lead to hepatocellular carcinoma.^188^ (2) It contributes to tumor invasion and metastasis. By remodeling the epithelial-mesenchymal transition (EMT), the SASP provides a conducive environment for tumor cell dissemination.^183,189,190^ Senescent fibroblasts secrete matrix metalloproteinase 3, which affects the morphological and functional differentiation of mammary epithelial cells, ultimately relaxing restrictions on cell migration/invasion.^191^ SnCs secrete a plethora of proangiogenic factors, thereby supporting tumor angiogenesis.^192^ Moreover, oxidative stress in the aging microenvironment contributes to tumor progression partly by promoting ROS-mediated platelet activation, which facilitates cancer cell protection and metastasis.^193,194^ Ferroptosis, which is induced by excessive ROS accumulation and lipid peroxidation, further amplifies oxidative stress and contributes to immune cell senescence in the tumor microenvironment.^195^ (3) It facilitates tumor evasion from immune surveillance, as the cytotoxicity of senescent NK cells and effector T cells is significantly reduced.^196^ It has been reported that senescence impedes tumor surveillance by DCs and the activation of OVA-specific T cells, ultimately resulting in suboptimal outcomes of OVA immunotherapy for melanoma.^197^ In another study, senescent DCs exhibited reduced secretion of IL-15, IL-18 and IFN-α, failing to activate NK cells and thereby exacerbating RMA-S lymphoma.^198^ However, some studies suggest that aging immune responses may have a suppressive effect on cancer.^199^ Compared with aged mice, young mice exhibit more rapid cancer growth and a deficiency in mature T and B lymphocytes.^200^ One of the antitumor mechanisms mediated by aging is the clearance of presenescent tumor cells through antigen-specific immune responses, thereby inhibiting tumor progression.^201,202^

Moreover, immunosenescence may have significant implications for cancer immunotherapy.^203^ Conventional tumor therapies, typically chemotherapy, radiotherapy, and surgery, can induce both spontaneous and therapy-induced senescence (TIS), accompanied by the accumulation of SnCs. Upon entering TIS, tumor cells undergo intrinsic reprogramming to acquire stem-like properties, thereby promoting tumor progression and contributing to therapeutic failure.^204^ In this process, the prominent feature is the accumulation of senescent and failing T cells. It has been reported that TIS suppresses CD8^+^ T cell activation, fostering an immunosuppressive microenvironment that contributes to poor treatment outcomes.^205^ Cancer immunotherapy focuses primarily on T cell-mediated approaches, and T cell senescence is a critical component of immunosenescence. Senescent T cells can recruit MDSCs and Tregs^205^ and further induce senescence in neighboring effector T cells,^206^ thereby establishing an immunosuppressive microenvironment that impedes effective antitumor immune responses. In the context where immune checkpoint blockade (ICB) has demonstrated remarkable efficacy in cancer immunotherapy, several studies have revealed the reduced effectiveness of anti-PD-1/PD-L1 therapy in aged mice and elderly patients,^207–209^ leading to treatment resistance. This evidence suggests a connection between immunosenescence and the diminished efficacy of ICB. Furthermore, the ICB response to shared antigens is largely mediated by memory T cells.^210^ These memory responses remain functional even in older patients, highlighting that immunological memory is not necessarily strongly impaired by aging.^8^ While cancer vaccines have emerged as a groundbreaking therapeutic approach in recent years, their clinical application faces a significant challenge: markedly reduced immunogenic responses in elderly patients relative to younger populations. With advancing age, the magnitude of the germinal center response and its output are impaired, coupled with spatial dysregulation of follicular helper T (Tfh) cells.^211,212^ For example, age-related upregulation of CXCR4 leads to mislocalization of Tfh cells to the dark zone, along with a significant reduction in the follicular dendritic cell (FDC) network area and a marked decrease in the number of plasma cells.^211^ Consequently, older adults generate lower antibody titers than younger individuals do, and these titers wane more rapidly, resulting in diminished vaccine efficacy in elderly individuals.^213,214^ Recent research suggests that tumors often present new antigens arising from mutations within individual cancer cells, and these neoantigens are typically recognized by naïve T cells.^215^ With age, the reduced pool of naïve T cells may impair the ability of the immune system to detect these antigens, creating what is referred to as “holes in the repertoire.” This limitation could reduce the effectiveness of immunotherapies targeting neoantigens.^203^ Despite this, advances in cancer mutation analysis and TCR sequencing have made it possible to assess whether an individual patient lacks the necessary TCRs to recognize crucial neoantigens.^216–218^ JAK-STAT dysregulation has also been implicated in promoting tumor immune evasion. Persistent STAT3 activation, in particular, suppresses cytotoxic responses and fosters MDSC accumulation, further exacerbating cancer risk.^33^ Moreover, the dysregulation of microRNAs (miRNAs) in the tumor microenvironment plays a crucial role in modulating immune responses and promoting tumor progression.^219^ Exosome-mediated lipid metabolic communication also plays a vital role in modulating the tumor microenvironment and promoting digestive system neoplasms.^220^

Chimeric antigen receptor T cell (CAR-T) immunotherapy, which primarily targets hematologic malignancies, including B cell acute lymphoblastic leukemia (B-ALL), is currently being explored for solid tumors.^221–223^ However, only a minority of patients achieve long-term disease remission.^224^ The inefficacy of CAR-T cell therapy can be attributed to multiple factors, among which T cell senescence and exhaustion play pivotal inhibitory roles.^225^ One piece of supporting evidence demonstrated that patients exhibiting lower T cell differentiation (characterized by naïve or early memory T cell predominance) consistently exhibited superior clinical responses.^226,227^ Currently, the definitions of T cell senescence and exhaustion are not fully distinguished. However, it is clear that T cell functionality indeed impacts the outcomes of CAR-T cell therapy. Specifically, the antitumor activity of adoptively transferred T cells relies on their memory and stem-like properties, whereas T cells from patients with poor therapeutic responses exhibit increased exhaustion and apoptosis markers.^228^ Furthermore, CD8^+^ T cells expressing senescence-associated molecules such as LAG-3 and PD-1-related molecules are associated with an unfavorable prognosis in CLL patients receiving CAR-T cell therapy.^228,229^ Moreover, the tumor-suppressive microenvironment harbors MDSCs and Treg cells, which are associated with inflammatory senescence and further promote T cell senescence.^184,230,231^ Conversely, CAR-T cell therapy can induce SASP-related cytokines, ultimately contributing to the formation of an inflammatory milieu.^232,233^ These findings suggest that postinfusion, CAR-T cells may also exacerbate treatment efficacy through inflammation-driven senescence.

### Other diseases

#### Age-related macular degeneration (AMD)

**Fig. 5:**
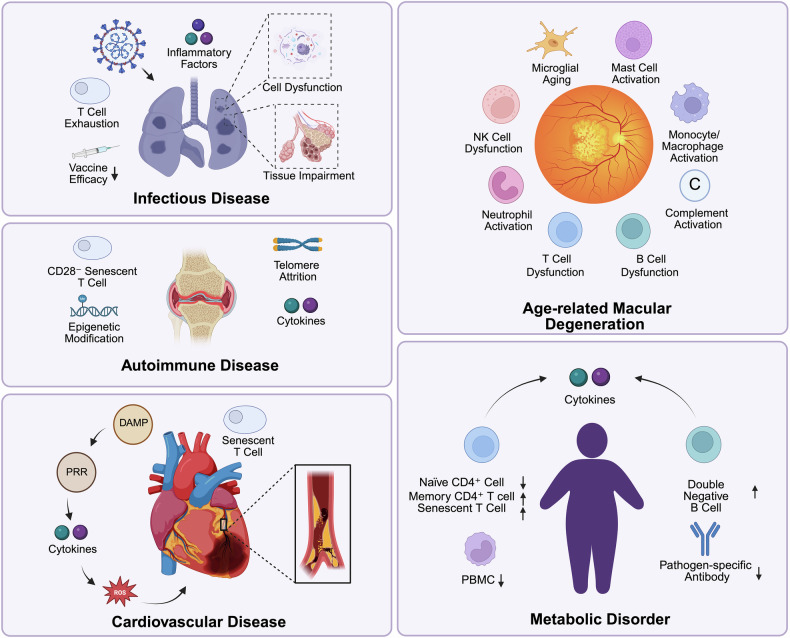
Other immunosenescence-related diseases. (1) Infectious diseases: Immunosenescence increases susceptibility to infections (e.g., SARS-CoV-2, CMV) due to PD-1/Tim-3 overexpression in T cells. Chronic inflammation impairs lung function, contributing to COPD and IPF. Weakened vaccine responses reduce protection in older adults. (2) Autoimmune diseases: Senescent CD28⁻ T cells disrupt immune tolerance, exacerbating RA. Telomere attrition and epigenetic changes sustain chronic inflammation and systemic complications. (3) CVD: Aging-induced inflammation and oxidative stress drive CVD. DAMPs activate PRRs, triggering cytokine and ROS production. Senescent T cells worsen vascular dysfunction. (4) AMD: Dysregulated immune responses and chronic inflammation damage the retina. Dysregulated microglia and NK, T, and B cells drive inflammation, whereas mast cells and monocyte/macrophage activation exacerbate retinal damage through proinflammatory cytokine release. Neutrophil NET formation and complement activation further impair the blood‒retinal barrier, accelerating AMD progression. (5) Metabolic disorders: Immunosenescence promotes inflammation in T2D and obesity. Increased memory CD4⁺ T cells and senescent T cells enhance cytokine production, whereas double-negative B cells expand, leading to enhanced proinflammatory responses and autoantibody secretion. Reduced PBMC function weakens immune defense, exacerbating metabolic dysfunction

Immunosenescence plays a significant role in the onset and progression of AMD by altering the inflammatory response of the immune system (Fig. 5). AMD is an ocular disease that causes blurred central vision, primarily due to aging-related damage to the macula.^234^ Immunosenescence promotes the development of AMD by causing low-grade chronic inflammation in the retina and choroid.^235,236^ As the eyes age, significant changes in the retinal pigment epithelium (RPE) occur. The accumulation of ROS, lipofuscin, and other byproducts gradually disrupts the metabolism and function of RPE cells, leading to progressive deterioration of retinal health and accelerating the progression of AMD.^236,237^ Elevated levels of proinflammatory cytokines from various immune cells during aging further contribute to ongoing damage to the retina and choroid. Microglial cells are innate immune cells that have self-renewal ability and neuroprotectivity in the normal retina. In AMD, aging microglia stimulate chronic low-grade inflammatory responses and exacerbate immune-mediated damage to the retina and RPE.^238,239^ Senescent macrophages in the eyes secrete proinflammatory cytokines via STAT3 signaling, aggravating retinal damage and promoting neovascularization, a hallmark of AMD.^240^ Mast cells also play an important role in the pathogenesis of AMD. During AMD, increased mast cell numbers and degranulation are observed, with increased production of proinflammatory mediators such as CXCL1.^241^ Neutrophils participate in retinal immune responses by releasing NETs. These NETs help clear aged blood vessels and immune cells, but excessive NET formation exacerbates retinal damage, especially in the context of excessive inflammation.^242^ T cells and B cells also play critical roles in immune responses in AMD. With aging, T cell tolerance decreases, leading to increased secretion of proinflammatory cytokines and exacerbated retinal damage.^243^ Complement system activation recruits immune cells and disrupts the blood‒retinal barrier, further promoting retinal inflammation and the progression of AMD.^244,245^

#### Metabolic disorders

Immunosenescence is closely linked to various metabolic disorders, particularly type 2 diabetes (T2D).^246^ The immune aging process results in functional alterations in immune cells, increasing the susceptibility of elderly individuals to metabolic dysfunction. Metabolic diseases such as T2D, obesity, and metabolic syndrome are associated with low-grade chronic inflammatory states (Fig. 5). This inflammation is called metabolic inflammation and is similar to the chronic inflammatory process that occurs during aging.^247^ Increased fat mass, metabolic dysfunction, and systemic inflammatory responses are key features of these metabolic diseases and can further exacerbate immune dysfunction associated with aging.^164^ In T2D, a hallmark of immune aging is a reduction in the proportion of naïve CD4^+^ T cells alongside increased memory CD4^+^ T cells and effector CD4^+^ and CD8^+^ T cells. These effector cells are the main producers of proinflammatory cytokines, including IFN-γ and TNF-α, leading to increased systemic inflammation.^164,248^ Additionally, an increase in the number of senescent T cells, including CD8^+^CD57^+^ and CD8^+^CD28^−^ T cells, is considered a predictor of hyperglycemia development in humans.^248^ Apart from adaptive immunity, aging induces defects in the function and activation of the innate immune system in T2D. In T2D patients, the phagocytic capacity and TLR responsiveness of peripheral blood mononuclear cells (PBMCs), which are closely related to endoplasmic reticulum stress and poor blood sugar control, are significantly impaired.^248,249^ Obesity is another metabolic disorder associated with metabolic inflammation and has a similar phenotypic spectrum as aging, such as dysfunctional mitochondria, weakened immunity, and elevated systemic inflammation.^250^ Obesity significantly impacts B cell function. Studies have demonstrated that B cells from obese individuals exhibit a reduced capacity to generate pathogen-specific antibodies.^251^ Furthermore, both obesity and aging drive the expansion of double-negative (DN) B cells (CD27⁻IgD⁻), which display a proinflammatory phenotype marked by autoimmune antibody secretion and upregulated expression of activation markers (e.g., CD11c and T-bet).^252^ Exposure to plasma from obese individuals promoted the apoptosis, DNA damage, and mitochondrial dysfunction of PBMCs, resulting in increased production of IL-1β and IL-8.^253^ Moreover, CD8^+^ T cells present an immunosenescent phenotype with reduced expression of CD28, indicating that chronic systemic inflammation in individuals with obesity promotes immune system dysfunction and aging.^253^

#### Infectious diseases

Immunosenescence in the elderly population is closely related to increased susceptibility to infectious diseases such as severe acute respiratory syndrome (SARS-CoV) and cytomegalovirus (CMV) infection (Fig. 5).^113^ As people age, the immune system undergoes functional decline. During CMV infection, the oligoclonal expansion of CMV-specific CD8^+^ T cells is inhibited in elderly individuals, which limits the ability to combat viral infection.^254^ The coronavirus disease 2019 (COVID-19) pandemic has starkly illustrated the clinical consequences of immunosenescence. Elderly patients infected with SARS-CoV-2 frequently experience rapid disease progression due to high expression of PD-1 and Tim-3 on CD8^+^ T cells and dysregulated innate immunity, culminating in cytokine storms dominated by IL-6 and granulocyte‒macrophage colony‒stimulating factor (GM-CSF).^255,256^ Moreover, immunosenescence compromises vaccine efficacy. Although mRNA vaccines (e.g., BNT162b2) partially mitigate age-related immune deficits by enhancing GC reactions and memory B cell generation, their protective efficacy against COVID-19 in older adults remains suboptimal.^257^ Strategic booster doses, however, significantly improved cross-protection against variants such as Omicron, highlighting the necessity of age-tailored vaccination protocols.^258^ Similarly, elderly individuals exhibit weaker antibody responses upon immunization with influenza and pneumococcal vaccines, underscoring the need for enhanced immunization strategies.^257,259^ Moreover, JAK3 mutations have been shown to lead to severe immunodeficiencies, whereas STAT3 mutations impair IL-17 production, which increases susceptibility to bacterial and fungal infections. These findings suggest that specific genetic mutations may contribute to the diminished ability of the immune system to effectively fight infections in elderly individuals, potentially compounding the challenges posed by immunosenescence.^34^

#### Respiratory diseases

In chronic obstructive pulmonary disease (COPD), aging-associated immune dysfunction exacerbates lung damage through multiple pathways: epithelial barrier integrity deterioration, impaired mucus clearance, and alveolar macrophages with low phagocytic capacity.^260,261^ These changes amplify inflammation triggered by environmental insults such as cigarette smoke, while accelerated cellular aging (e.g., telomere shortening in alveolar cells) further drives oxidative stress and apoptosis.^262^ A parallel mechanism is observed in idiopathic pulmonary fibrosis (IPF), where telomere attrition in lung fibroblasts, combined with chronic inflammation and TGF-β pathway activation, promotes excessive collagen deposition and irreversible structural damage.^263,264^

#### Autoimmune diseases

Rheumatoid arthritis (RA) is a chronic inflammatory disorder characterized by symmetrical and destructive inflammation of joints and other organs/tissues. As individuals age, the immune system shifts toward a more proinflammatory state, marked by the accumulation of CD28^-^ T cells. These senescent T cells disrupt immune tolerance, promote autoreactivity against self-antigens, and exacerbate disease severity, particularly in RA patients with extra-articular manifestations (Fig. 5).^265^ In addition, genetic factors, such as STAT3/STAT4 polymorphisms, contribute to the development of autoimmune diseases such as RA by impairing immune tolerance and increasing susceptibility to autoreactivity.^33^ Increased telomere attrition in these T cells exacerbates the inflammatory state and is associated with the development of cardiovascular disease in RA patients, indicating the systemic effects of immunosenescence.^262,266^ The complex interplay between immunosenescence and autoimmunity highlights the importance of further research and the development of novel therapeutic approaches to treat autoimmune diseases in the elderly population.

#### Cardiovascular diseases (CVDs)

Aging is the most significant risk factor for CVD, which remains the leading cause of death worldwide.^267^ CVD encompasses a range of heart and vascular diseases, which are associated with the biological process of aging, the loss of homeostasis, and increased morbidity and mortality rates (Fig. 5).^268^ Inflammaging is a key risk factor for CVD and involves elevated levels of proinflammatory cytokines, leading to endothelial damage, impaired vascular remodeling,^269^ and atherosclerosis.^270^ These inflammatory molecules are secreted primarily by senescent T cells and proinflammatory macrophages.^271,272^ This reflects the body’s inability to properly regulate immune responses during aging, driving tissue dysfunction and pathological alterations. During cardiac stress, ischemic injury, and metabolic syndrome in the cardiovascular system, necrotic cells release DAMPs, which are recognized by pattern recognition receptors on innate immune cells, triggering strong inflammatory responses.^273^ This leads to the secretion of proatherosclerotic cytokines, ROS, and reactive nitrogen species (RNS), amplifying oxidative stress. These cytokines also stimulate the proliferation of vascular smooth muscle cells (VSMCs) and the accumulation of oxidized low-density lipoprotein (LDL) particles, which are then captured by foam cells in vessel walls.^270^ Senescent T cells, particularly cytotoxic CD8^+^ T cells, also contribute to the pathophysiology of CVD. The expansion of CD8^+^CD28^−^ T cells is a risk factor for vascular dysfunction.^274^ Studies have shown that the expansion of peripheral late-differentiated CD4^+^CD28^−^ T cells that produce IFN-γ after persistent antigenic stimulation is observed in unstable angina.^275^ In older men, CMV infection-related atherosclerosis may be mediated by an increased proportion of memory CD4⁺ T cells.^276^

## Therapeutic targets in immunosenescence

**Fig. 6:**
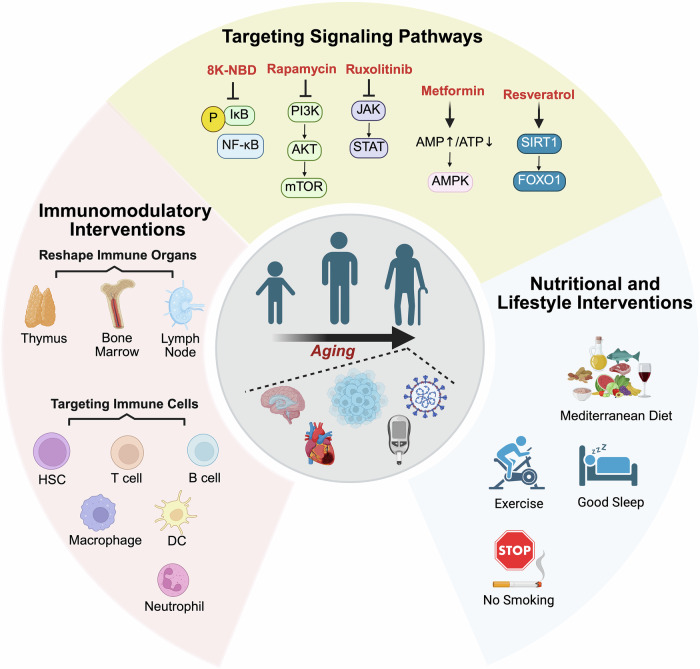
Therapeutic strategies related to immunosenescence. The three types of therapeutic measures mentioned in the review are as follows: (1) Immune intervention, which is mainly divided into interventions targeting immune organs and immune cells. (2) Targeting signaling pathways related to aging; slowing the immune aging process by downregulating NF-κB, mTOR, and JAK-STAT; and upregulating AMPK, SIRT1 and other signaling pathways. (3) Nutritional and lifestyle intervention strategies

The study of aging has long been a primary objective for scientists. Over the past few decades, the mechanisms and pathways underlying this critical aspect of immune senescence have been explored via advanced biological techniques and genetic tools. Notably, these mechanisms and pathways represent essential targets for interventions aimed at mitigating immunosenescence. Consequently, in this chapter, we summarize the current status of interventions aimed at combating or delaying immunosenescence. The emerging strategies include targeted therapies, immune interventions, and lifestyle modifications, among which there are several promising results (Fig. 6).

### Targeting signaling pathways

#### NF-κB signaling pathway

Persistent NF-κB activation drives the SASP^267^. A study demonstrated that sustained inhibition of NF-κB for a period of two weeks can reverse tissue characteristics and the overall gene expression program.^277^ Direct inhibitors of the IKK/NF-κB pathway may confer clinical benefits for degenerative changes associated with both progeroid syndrome and normal aging.^10,278^ Genetic suppression of the IKK/NF-κB pathway, achieved through the deletion of one p65 allele, or pharmacological intervention via IKK inhibitors, such as 8K-NBD, has been shown to delay the onset and mitigate the severity of aging symptoms and age-related pathologies in the nervous system of murine models^10,279^. Bortezomib was demonstrated to inhibit the proteolysis of IκB, thereby preventing the activation of NF-κB;^280^ this represents a promising and innovative approach to delay aging (Table 1).^281^ Additionally, fisetin promotes the synthesis of the antioxidant glutathione and suppresses the activity of proinflammatory factors, including TNFα, IL-6, and the transcription factor NF-κB.^282,283^ Several natural phytochemicals, such as curcumin, have also been shown to inhibit NF-κB nuclear translocation while simultaneously activating Nrf2, an antioxidative pathway.^284^ The loss of PTEN activates the AKT/NF-κB pathway, thereby promoting alveolar epithelial cell senescence and the release of SASP^285,286^^.^ EF24, a notable derivative of curcumin, has been demonstrated to increase PTEN expression and subsequently inhibit the NF-κB pathway (Table 1).^287^ Resveratrol activates SIRT1, leading to the deacetylation of the p65 subunit of NF-κB, thereby reducing its transcriptional activity.^288^ Notably, clustered regularly interspaced short palindromic repeats (CRISPR)-Cas is currently recognized as one of the most powerful gene-editing tools available. The hyperactivation of the gap junction protein connexin 43 (Cx43) increases the expression of p53, p16INK4a, and NF-κB, which are positively correlated with aging. CRISPR/Cas9-mediated downregulation of Cx43 inhibits the transition of chondrocytes into a senescent state.^289^

**Table 1:** Interventions related to signaling pathways

Related signaling pathway	Potential interventions	Representative drugs	Condition	Reference
NF-κB signaling pathway	NRF2 promoter/activator, inhibits IkB-α phosphorylation, degradation, and p65 nuclear translocation	Sulforaphane (SFN)	Diabetic neuropathy, Traumatic brain injury, Spinal cord injury	^484–486^
	Inhibits the proteolysis of IκB and prevents the activation of NF-κB	Bortezomib	Breast cancer, Prostate cancer, Colon cancer	^487–489^
	Enhances PTEN expression and inhibits the NF-κB	EF24	Idiopathic pulmonary fibrosis	^287^
mTOR signaling pathway	Inhibition of mTORC1 activity	Rapamycin	Alzheimer’s Disease, Parkinson disease	^490,491^
	Inhibits mTORC1 and mTORC2	PP242	Esophageal squamous cell carcinoma, Ovarian	^299,492,493^
JAK-STAT signaling pathway	Inhibits Janus kinase (JAK) 1 and 2	Ruxolitinib	Myelofibrosis, Glucocorticoid-Refractory Chronic Graft-versus-Host Disease	^494,495^
AMPK signaling pathway	Reduces the ADP/ATP and AMP/ATP ratios and activates AMPK	Metformin	utchinson-Gilford progeria syndrome	^311^
Melatonin signaling pathway	Antioxidant function, Stabilization of mitochondrial function and enhancement of autophagy, Anti-inflammatory effects	Melatonin	Alzheimer’s disease, Parkinson’s disease, Huntington’s disease	^320,496^
Sirtuin signaling pathway	Modulates the deacetylation state of the core autophagy protein ATG9A by activating SIRT1, ameliorates oxidative stress by increasing SIRT1 expression	Resveratrol	Age-related hearing loss, Alzheimer’s disease, Obesity	^497,498^
PI3K/AKT signaling pathway	Targets Bcl-2 family members, hypoxia-inducible factor-1α (HIF-1α), as well as anti-apoptotic PI3K/AKT and p21 signaling pathways	Quercetin	Obesity-induced glucose intolerance and insulin resistanc	^337^
FOXO4-p53 signaling pathway	Disrupt the FOXO4-p53 interaction, thereby reducing senescence-induced FOXO4 activity and selectively targeting SnCs for p53-dependent apoptosis	FOXO4-DRI	Tissue Homeostasis in Response to Chemotoxicity and Aging	^346^

#### mTOR signaling pathway

There is substantial evidence demonstrating that the mTOR signaling pathway is a critical target for antiaging interventions. Inhibition of mTORC1 promotes autophagy, which facilitates the clearance of unwanted cytoplasmic proteins and reduces the accumulation of toxic metabolites, thereby mitigating cellular stress and extending lifespan.^290,291^ Another proposed mechanism is that mTOR regulates the crosstalk between mitochondria and the nucleus, stabilizing the key protein Clk-1, which is essential for mitochondrial signaling communication.^292–294^ The first-generation mTOR inhibitor rapamycin has been shown to extend lifespan across various organisms, making it the only known pharmacological agent that directly modulates aging (Table 1).^295–297^ However, Cloughesy et al. reported that while rapamycin reduces the proliferation of glioblastoma, its effects are not sustained.^298^ Consequently, the primary goal of second-generation mTOR inhibitors is to simultaneously target both the mTORC1 and mTORC2 signaling pathways, as well as their feedback loops, which were not addressed by first-generation inhibitors.^299^ By providing more comprehensive inhibition of the mTOR signaling pathway, second-generation mTOR inhibitors, including PP242, KU0063794, AZD3147, and eCF309, have shown promising results in preclinical and clinical trials.^300,301^ The most recent advancement, RapaLink-1, structurally resembles rapamycin and binds to mTOR, effectively inhibiting mTORC1.^302^ Compared with rapamycin, RapaLink-1 has been reported to exert a stronger inhibitory effect on T cell proliferation.^302^ Phase IIa and IIb clinical trials investigating the combination of the mTORC1 inhibitors RTB101 and RAD001 revealed that this regimen could reduce immunosenescence and enhance the response to influenza vaccination.^29,303^ However, the phase III trial of RTB101 alone failed to achieve the desired outcomes.^303^

#### JAK-STAT signaling pathway

During the aging process, sustained activation of the JAK-STAT signaling pathway induces the expression of the SASP, promoting cellular senescence and apoptosis, as well as impairing the function of T cells, B cells, and macrophages. Targeting the JAK-STAT signaling pathway can alleviate chronic inflammation, improve immune cell function, and delay the onset of aging-related phenotypes.^34,304^ Notably, the JAK pathway is more active in the adipose tissue of aged animals than in that of their younger counterparts. Compared with that in younger mice, the efficacy of the JAK inhibitor ruxolitinib in aged mice is superior, manifesting in the clearance of SnCs, enhancement of physical performance, and maintenance of adipose tissue homeostasis (Table 1). This evidence suggests that JAK inhibitors confer beneficial effects by modulating SnCs.^304,305^ The percentage of SnCs was significantly reduced in mice treated with the JAK inhibitor NVP-BSK805 in combination with docetaxel, thereby enhancing the antitumor response to docetaxel.^306^ Additionally, JAK inhibitors may promote hair growth by stimulating the activation and proliferation of stem cells.^307^ The underlying mechanism could be leveraged to directly target tissue stem cells and their respective niches.^307^ The administration of JAK inhibitors leads to a significant increase in satellite cell populations, facilitates robust muscle regeneration, and elevates functional capabilities, thereby presenting a viable and innovative therapeutic strategy for addressing muscle wasting conditions.^308^

#### AMPK signaling pathway

Metformin is a well-known activator of AMPK that functions by reducing the ADP/ATP and AMP/ATP ratios (Table 1).^309^ This reduction is achieved through partial inhibition of Complex I of the mitochondrial ETC. Consequently, this inhibition leads to direct or indirect activation of AMPK.^310^ In age-related diseases, metformin beneficially enhances mitochondrial function, increases Nrf2 activity, induces autophagy, or ameliorates accelerated aging defects in Hutchinson–Gilford progeria syndrome (HGPS) cells by altering gene splicing through its activation mechanisms.^311^ Metformin can also mitigate age-related hearing loss and neurodegeneration symptoms in D-galactose-induced aging rats by modulating the AMPK/extracellular signal-regulated kinase 1/2 (ERK1/2) signaling pathway.^312^ In addition, we cannot overlook the role that resveratrol plays in the AMPK pathway. It has been reported in the literature that resveratrol can prevent oxidative stress-induced aging and proliferation damage by activating the AMPK/FOXO3 signaling pathway.^313^ Oleanolic acid induces autophagy and apoptosis in colon cancer cells by modulating the AMPK-mTOR signaling pathway, thereby exerting therapeutic effects against colorectal cancer.^314^

#### Melatonin signaling pathway

The mechanisms by which melatonin-related signaling pathways exert their antiaging effects can be summarized as follows (Table 1): (1) Antioxidant function. Melatonin can directly scavenge ROS through its electron-rich indole ring, and it can also induce antioxidant enzymes such as GPX, SOD, and catalase to achieve antioxidative effects. Furthermore, melatonin enhances the efficacy of antioxidant vitamins. (2) Stabilization of mitochondrial function and enhancement of autophagy. As previously mentioned, mitophagy is a crucial step in protecting aging cells from damage caused by endogenous waste stress.^315^ Melatonin enhances mitophagy, which can alleviate the accumulation of dysfunctional mitochondria and restore mitochondrial quality.^316,317^ (3) Anti-inflammatory effects. Melatonin exerts its neuroprotective effects by inhibiting the release of cytochrome and the activation of caspases, as well as negatively regulating the production of proinflammatory cytokines.^318^ Early melatonin intervention disrupts the vicious cycle of Aβ-ROS-induced neuroinflammation in AD and ameliorates metabolic inflammation in T2D.^47,319^ The outcomes of clinical trials in these areas have been highly encouraging, underscoring its efficacy. However, while preclinical investigations of cardiovascular diseases have yielded promising results, melatonin has not met anticipated efficacy benchmarks in translational studies involving patients with established cardiovascular conditions.^320,321^ Nonetheless, it has shown notable protective effects against cardiovascular risk factors.^321^ Consequently, a deeper exploration of its mechanistic pathways and therapeutic potential is warranted, aiming to elucidate novel strategies and substantiate evidence for the prevention and management of these diseases.

#### Sirtuin signaling pathway

Sirtuins are a family of NAD^+^-dependent deacetylases that serve as crucial regulators in delaying cellular senescence and extending organismal lifespan.^322^ With advancing age, there is a notable decline in sirtuin activity, particularly in SIRT1 and SIRT3.^323,324^ The overexpression of SIRT1 has been demonstrated to inhibit the aging of nucleus pulposus cells, promote cell proliferation, and suppress apoptosis.^325^ Moreover, the activation of SIRT1 also inhibits the senescence of dermal fibroblasts induced by ultraviolet irradiation.^326^ On the basis of the aforementioned research, increasing interest has been directed toward various NAD^+^ precursors or small-molecule activators, which aim to increase the activity of sirtuins.^323^ Intriguingly, the first SIRT1 activator to capture widespread attention was resveratrol, which exerts its effects through several mechanisms (Table 1).^327^ For example, it can modulate the deacetylation state of the core autophagy protein ATG9A by activating SIRT1, thereby delaying age-related hearing loss.^328^ Additionally, resveratrol improves motor function in senescence-accelerated mice by attenuating the negative regulation of insulin and apoptotic signaling through the SIRT1/FOXO1 pathway.^329^ These findings underscore the capacity of resveratrol to exert its antiaging effects across diverse biological pathways.

Notably, SIRT1, which serves as a pivotal modulator in the promotion of healthspan, captures scientific intrigue largely because of its ability to orchestrate a multitude of signaling cascades. SIRT1 interacts with the RelA/p65 subunit of NF-κB, leading to the deacetylation of lysine residues on this subunit. This interaction ultimately diminishes the transcriptional activity of the NF-κB complex, and as a result, SIRT1-mediated deacetylation serves to inhibit NF-κB signaling.^330^ Concerning another significant pathway that promotes aging—the mTOR pathway—SIRT1 can obstruct mTOR signaling, thereby rectifying autophagy impairments induced by oxidative stress and consequently enhancing the survival rate of embryonic stem cells.^331^ In alignment with this, SIRT1 and AMPK can reciprocally enhance each other’s activity. SIRT1 deacetylates LKB1, thereby activating AMPK and inhibiting senescence^332^. Conversely, AMPK can increase intracellular NAD^+^ levels, which in turn boosts SIRT1 activity, ultimately delaying the aging process of chondrocytes in osteoarthritis.^333,334^

#### Other targets of signaling pathways

Dasatinib, a common drug for targeting SnCs, is a second-generation tyrosine kinase inhibitor that can downregulate the expression of senescence-associated biomarkers, including β-galactosidase, p16, and p21.^335^ Quercetin, which targets the Bcl-2 family member hypoxia-inducible factor-1α (HIF-1α) and the antiapoptotic PI3K/AKT and p21 signaling pathways, has demonstrated synergistic effects (Table 1).^336^ This combination has been shown to mitigate adipose tissue inflammation and cellular senescence in aged mice, concurrently enhancing systemic metabolic performance.^337,338^ It has been shown to prevent the progression of age-dependent intervertebral disc degeneration,^339^ reduce intestinal SnCs and excessive inflammation in aged mice,^340^ and effectively induce apoptosis, thereby eliminating SnCs and the SASP burden in adipose tissue.^337^ Additionally, navitoclax (also known as ABT-263) is a drug classified as a “Bcl-2 family inhibitor.” It specifically inhibits the antiapoptotic proteins Bcl-2, Bcl-xL, and Bcl-w within the Bcl family and induces apoptosis through the activation of caspase signaling pathways.^341^ For example, the oral administration of Navitoclax in mice subjected to sublethal irradiation or natural aging effectively depletes senescent hematopoietic stem cells and senescent muscle stem cells, thereby rejuvenating stem cell function.^342^ However, its side effects, such as transient thrombocytopenia and neutropenia, remain obstacles to clinical translation.^342–344^ It has been reported that p53 is a senescence marker and that the p53 signaling pathway regulates cellular senescence.^181^ In another study, FOXO4 was found to be elevated in SnCs and contributed to the senescence process.^345,346^ FOXO4-DRI, a cell-permeable peptide, disrupted the FOXO4-p53 interaction, thereby reducing senescence-induced FOXO4 activity and selectively targeting SnCs for p53-dependent apoptosis (Table 1).^346^ FOXO4-DRI inhibits renal tubular senescence and restores renal function in vivo^347^. Additionally, it effectively eliminates senescent chondrocytes in vitro and ameliorates pathological changes and collagen deposition in the lungs of mice with pulmonary fibrosis.^348,349^

### Targeting immune cells

#### Hematopoietic stem cells

Dysfunction of HSCs may serve as a fundamental basis for the aging of the myeloid and lymphoid lineage systems.^350^ For example, aged HSCs exhibit a diminished capacity to adhere to stromal cells, which may perturb the interactions between stem cells and the hematopoietic niche, ultimately leading to alterations in the functionality of aging HSCs.^351,352^ Therefore, the underlying causes of stem cell aging are likely to represent potential therapeutic targets. One contributing factor to the changes observed in senescent stem cells may be the age-dependent acquisition of defects in telomeres, genomic DNA, or mitochondrial DNA.^353–356^ During the process of replicative aging of HSCs in both humans and mice, telomeres progressively shorten.^357^ The function of telomerase in HSCs is to counteract the rate of telomere shortening during cell division, thereby preventing premature telomere attrition and extending the replicative capacity of HSCs.^357^ The in vitro culture of telomere-binding protein protection of telomeres 1 (Pot1) in HSCs has been demonstrated to maintain self-renewal capacity and regulate the activity of HSCs in elderly individuals, which has significant implications for the ex vivo cultivation of human HSCs.^358^ Similarly, gene therapy employing adeno-associated virus (AAV) 9 vectors to overexpress telomerase reverse transcriptase (TERT) has been demonstrated to alleviate age-related telomere damage and delay senescence in mouse models of cancer resistance.^359^

The diminished efficacy of HSCs can be attributed not only to telomere shortening but also to an increased risk of DNA damage^360^ and elevated levels of ROS production.^361^ In the context of ataxia-telangiectasia mutated (ATM) deficiency, treatment with the permeable thiol antioxidant N-acetyl-l-cysteine (NAC) was shown to restore the reconstitution capacity of HSCs, thereby preventing bone marrow failure.^361^ Furthermore, inhibition of the upregulated tumor suppressors p16^INK4a^ and p19^ARF^ in the context of ATM deficiency has been demonstrated to restore the replicative function of HSCs.^361^ However, p16^INK4a^ does not universally induce HSC senescence in all contexts, and its role in maintaining HSC homeostasis has not been reported in other studies.^362^ A new study revealed that depleting aged murine HSCs with myeloid-biased outputs (my-HSCs) via antibodies such as anti-CD150 can restore features of a younger immune system in mice. This intervention increased naïve T cells and mature B cells in aged mice while reducing the levels of inflammatory markers.^363^ Interestingly, in aging tissues, the inhibition of key molecules with high activity in oxidative stress and strong ROS inducers, such as p38, can restore the function of HSCs.^350,364^ SIRT3, a prominent member of the sirtuin family, enhances antioxidant activity and exerts an inhibitory effect on ROS. Upregulation of SIRT3 rescues functional impairments in aged HSCs.^365^ Notably, mTOR and cell division control protein 42 (CDC42) are closely associated with the aging process and have been identified as promising targets for rejuvenating HSCs.^295,366–368^ The small-molecule CDC42-targeting drug CASIN has significant potential in mobilizing murine HSCs, thereby contributing to overall lifespan extension.^369,370^ Furthermore, targeted therapies against these proteins may not only lead to the rejuvenation of HSCs but also potentially induce systemic rejuvenation of the entire organism.^371,372^ Inflammation associated with aging induces the expression of interleukin-27 receptor α (IL27Ra) on the surface of HSCs through the TNF-α-ERK-ETS1 signaling pathway, leading to HSC senescence.^373^ The pharmacological targeting of key molecules within this signaling pathway may provide new therapeutic avenues for combating immune aging and related diseases caused by HSC senescence.^373^ In summary, the concept of revitalizing tissue-specific stem cells to restore tissue function and, ultimately, rejuvenating the whole organism represents a promising avenue worthy of further exploration. Hematopoietic stem cell transplantation is a treatment method with promising therapeutic outcomes. An intervention involving the shared circulation of blood between two individuals of different ages has been reported to rejuvenate the state and functionality of stem cells in the brain, liver, and muscle tissues of older individuals.^374–376^ However, subsequent studies demonstrated that this rejuvenation effect is attributed to noncellular factors present in the blood rather than the cells themselves.^375,377,378^

#### T cell

**Fig. 7:**
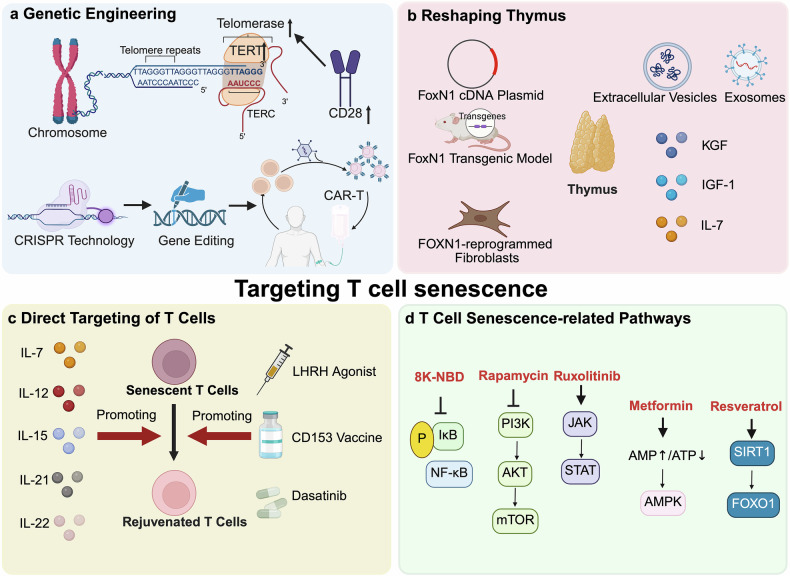
Targeting T cell senescence. **a** At the genetic level, modifying T cell functionality or engineering chimeric antigen receptor (CAR)-T cells with CD28 may prolong the duration of adoptive cell therapy by mitigating T cell senescence. **b** Thymic rejuvenation through thymic remodeling supports T cell development and maturation. **c** By directly targeting senescence-associated molecules in aged T cells, this approach can reverse T cell senescence. **d** Regulating signaling pathways associated with T cell senescence

In recent decades, it has become increasingly recognized that T cell senescence is a significant aspect of the aging process. The development of targeted therapies specifically aimed at modifying senescent T cells remains an ongoing area of research for the treatment of diseases associated with immune aging. Like HSCs, T cells are also affected by telomere shortening. On this basis, a specific subset of T cells with elevated expression of CD28 (a costimulatory molecule required for T cell activation) is capable of maintaining robust telomerase activity upon stimulation^379^. Therefore, the methods previously described for telomere extension and telomerase-mediated approaches can still reactivate T cells (Fig. 7). Furthermore, an intriguing discovery has been made that naïve and central memory T cells can elongate their telomeres by acquiring telomeric components from antigen-presenting cells (APCs) through immunological synapses.^380^ This mechanism may also represent a potential breakthrough for extending T cell telomeres. By employing the safe and feasible gene-editing tool CRISPR/Cas9 to engineer T cells, this method can mitigate exhaustion-inducing signaling pathways, preserve antitumor activity, and increase the in vivo persistence of CAR-T cells, thereby further overcoming limitations in cancer therapy (Fig. 6).^225,381,382^ The incorporation of additional costimulatory domains (such as 4-1BB or CD28) into engineered T cells enhances the persistence of CAR-T cells.^383,384^

Direct targeting of pathogenic T cells constitutes a widely investigated therapeutic strategy (Fig. 7). For example, the proliferation and functional restoration of T cells can be stimulated through the use of cytokines. IL-7 modulates the size of the peripheral T cell pool and plays a pivotal role in regulating T cell homeostasis. In the context of hematopoietic cell transplantation, the administration of exogenous IL-7 can facilitate T cell reconstitution, thereby promoting the homeostatic proliferation of T cells and conferring antiapoptotic effects on peripheral T cells.^385,386^ In addition to IL-7, several other cytokines that play pivotal regulatory roles in T cell regeneration have been identified in preclinical studies. IL-12 acts as an enhancer cytokine, potentiating the IL-2 and IL-7 signaling pathways, thereby sustaining thymic T cell function and supporting their development during aging.^387^ IL-15 is critical for enhancing the development and functional efficacy of CD8^+^ T cells.^388,389^ IL-21 has demonstrated efficacy in promoting the reconstitution of the peripheral naïve T cell compartment across diverse models of immune injury.^389^ Additionally, IL-22 facilitates the development of thymic-derived peripheral T cells.^390^ Together, these cytokines collectively contribute to the maintenance and restoration of T cell homeostasis, underscoring their therapeutic potential in immune regeneration and repair processes.

On the basis of the previously discussed evidence, keratinocyte growth factor (KGF) has been demonstrated to significantly enhance thymic regeneration. The therapeutic administration of recombinant KGF expedites the recovery of the peripheral T cell compartment and replenishes T cell populations subsequent to immune insults such as irradiation, cyclophosphamide administration, and dexamethasone treatment. In clinical practice, the utilization of KGF holds promise for the efficient reconstitution of the T cell compartment, thereby reinstating the ability to elicit a potent adaptive immune response.^391,392^ In an experimental study evaluating the effects of KGF therapy on thymic architecture and T cell immune reconstitution following myeloablative total body irradiation and autologous peripheral blood progenitor cell (PBPC) transplantation in healthy rhesus macaques, KGF treatment significantly increased de novo T cell production and the recovery of naïve T cells. These findings underscore the potential of KGF to promote robust thymic regeneration and immune restoration in the context of intensive conditioning regimens.^393^ Furthermore, the combined administration of KGF and androgens resulted in the accelerated reconstitution of naïve CD4^+^ and CD8^+^ T cells with a diverse Vβ repertoire, significantly contributing to the restoration of peripheral T cell populations.^394^

The capacity of sex hormones to influence lymphocyte development has been recognized for decades. In particular, the ability of estrogen to suppress postnatal thymocyte development and T cell production has been well-documented.^395^ Sex steroid ablation has been widely demonstrated to be beneficial for T cell reconstitution. A study showed that castration in 9-month-old mice rapidly reversed thymic involution, restoring the numbers of CD4^+^ and CD8^+^ cells to those of 2-month-old mice.^396^ Therapeutically, strategies that target sex steroid-related pathways, such as luteinizing hormone-releasing hormone (LHRH), or directly block sex steroid receptors can also achieve similar effects. A nonrandomized trial indicated that administration of the LHRH agonist goserelin (Zoladex) increased the numbers of various T cell subsets, particularly naïve CD4^+^ T cells, and improved the restoration of TCR repertoire diversity.^397^ LHRH agonists represent an effective and rational strategy for enhancing thymic function, not only in immunocompromised patients but also during normal aging.^398^ AR inhibitors and LHRH antagonists, which have the advantage of bypassing the sex steroid surge observed with LHRH agonists, may offer a greater opportunity for T cell reconstitution.^399^ However, it is worth considering that the regenerative effects of sex steroid inhibition on T cell development may be sustained only if sex steroid levels remain suppressed and that sex may also influence the efficacy of these interventions.^400^

The CD153 vaccine has been demonstrated to attenuate the presence of CD153^+^ senescent T cells within the adipose tissue of diet-induced obese mice through the induction of mouse IgG2 antibodies following administration.^401^ Additionally, the adoptive transfer of in vitro expanded or genetically engineered CD4^+^ T cells has emerged as a promising therapeutic approach to rejuvenating immune function in the context of immunosenescence. Notably, specific pharmacological interventions have shown potential in mitigating immunosenescence via T cell modulation. For example, dasatinib, a senolytic agent that inhibits tyrosine kinase (TK) activity and regulates the SASP, has been shown to promote the differentiation of CD4^+^ T cells toward a more juvenile phenotype.^402^ Furthermore, the mitochondrion-targeted antioxidant plastoquinonyl-decyltriphenylphosphonium has been shown to increase the intrathymic CD4^+^ T-to-CD8^+^ T cell ratio and prolong lifespan in diverse species.^403^ In elderly populations, metformin administration may ameliorate age-related impairments in autophagy and its downstream effects on CD4^+^ T cells.^404^ Recent studies have reported that micro/nanomaterial-based artificial antigen-presenting cell platforms can induce T memory stem cells in vitro and sustain long-term T cell immunity, thereby providing durable immune competence in elderly individuals.^405^

#### Other immune cells

Experimental evidence indicates that mice lacking MyD88, a critical adapter molecule for TLR signaling, lack ABCs and that pharmacological inhibition of this signaling axis effectively impedes the commitment of B cells to the ABC lineage.^116,406–408^ The downstream mediators of TLR7 and TLR9 signaling include IFN-γ and IL-21. In the context of IFN-γ deficiency, IL-4 exerts a suppressive effect on ABC differentiation, and targeted inhibition of IFN-γ or IL-21 represents a potential therapeutic avenue.^406^ Several studies have shown that follicular B (FO B) cells can serve as progenitors for ABCs, and their findings further revealed that neither MHC-II-deficient nor CD40-deficient FO B cells are capable of generating ABCs.^408–410^ Moreover, CD154-deficient mice exhibit an age-dependent failure to develop natural ABCs. Another recently characterized age-associated B cell population is aged adipose B cells (AABs), which localize to fat-associated lymphoid clusters and display a phenotypic profile distinct from that of ABCs.^411^ The expansion of AABs is mediated by Nlrp3 inflammasome activation.^411^ Therapeutic strategies involving the blockade of IL-1 signaling to inhibit Nlrp3-dependent B cell accumulation or the targeted depletion of B cells within adipose tissue via anti-CD20 antibodies have been shown to mitigate metabolic dysfunction in aged AT.^411^ CASIN, which is utilized for the treatment of aged HSCs, has also been shown to restore B cell populations and extend the posttransplant lifespan.^366^ Exogenous supplementation with spermidine can effectively promote the translation of TFEB, a transcription factor involved in autophagosome and lysosome biogenesis, through hypusinated EIF5A. This process reverses the age-dependent decline in the EIF5A-TFEB-autophagy axis and restores B cell function.^412^

Therapeutic blockade of NKG2A, an inhibitory receptor that modulates NK cell cytotoxic activity, enhances NK cell-mediated immunosurveillance and significantly diminishes senescent cell accumulation in aged murine models.^413,414^ The development and functionality of NK cells are regulated by a variety of miRNAs. Among these, the expression of miR-181a-5p notably decreases with age.^128^ This reduction is partially mediated through the upregulation of NLK and BCL2, which may contribute to the functional impairment observed in NK cells during aging.^128,415^ Consequently, the targeted delivery of miR-181a-5p to NK cells represents a clinically significant avenue of research aimed at enhancing the maturation and functional efficacy of NK cells in elderly individuals. Furthermore, it is well established that immunosenescence and inflammation are intricately interconnected. Immune cells that mediate the initiation and progression of inflammatory responses consequently represent promising therapeutic targets for intervention in age-related immune dysfunction. In monocyte/macrophage lineages, pharmacological modulation via the metabolic agent metformin achieves dual therapeutic effects by impairing monocyte-to-macrophage differentiation, consequently attenuating age-related macrophage senescence and systemic immune dysfunction.^416^ Pharmacological intervention through p38-MAPK signaling pathway inhibition or COX-2 suppression has demonstrated efficacy in restoring monocyte functional competence.^417^ Notably, cytokine-based intervention with IL-4 administration confers cytoprotective effects on macrophages while enhancing their survival capacity, ultimately ameliorating age-associated physiological decline and increasing health span parameters in geriatric mouse cohorts.^418^ Interestingly, recent studies have revealed that immunoglobulin G (IgG) induces both senescence and a proinflammatory state in macrophages from mice and humans. An antisense oligonucleotide targeting Fcgrt (encoded by FcRn depletion, which reduces tissue IgG levels) decreases IgG levels and reduces senescence-associated marker levels.^419^ Moreover, transplantation of eosinophils from young donors into aged recipient mice transiently reversed age-associated alterations in the HSC pool, leading to measurable improvements in physiological and immune health parameters that were partially mediated by eosinophil-derived IL-4.^420^ Brahmakshatriya and colleagues demonstrated that adoptive transfer of ex vivo activated bone marrow-derived DCs into aged mice enhances postimmunization GC formation and Tfh cell responses.^421^ Importantly, a separate study revealed that the reduction in the number of type 2 conventional dendritic cells (cDC2s) within the DC compartment may account for the attenuated vaccine efficacy observed in aged populations.^422^ Topical administration of the TLR7 agonist imiquimod was shown to increase antigen-bearing cDC2 populations, resulting in partial restoration of vaccination efficacy through targeted phenotypic restoration.^422^

### Reshaping immune organs

#### Thymus

Thymic involution is indeed one of the key characteristics of immunosenescence. As people age, the thymus gradually atrophies, leading to a decrease in T cell production, which in turn affects the function of the immune system. Therefore, thymic regeneration is considered a potential strategy to combat immunosenescence. Thymic epithelial cells (TECs) primarily maintain thymic development to ensure their integrity and support T cell development (Figs. 6, 7). Therefore, it is feasible to develop a developmental niche within the thymus by amplifying TECs and to concentrate on the interplay between TECs and thymocytes to increase the flux of thymopoiesis.^423,424^ Importantly, Forkhead box N1 (FOXN1) regulates the formation and expansion of TECs, making it a key regulatory factor in thymic development and even regeneration.^425^ Therefore, for the rejuvenation of the aging thymus, our strategies focused on the FOXN1-TEC axis, such as (1) the enhancement of thymic rejuvenation by the exogenous expression of FoxN1 in TECs via FoxN1 cDNA plasmids and FoxN1 transgenic models;^426^ (2) the implantation of FOXN1-reprogrammed embryonic fibroblasts (FREF) into the senescent native thymus to restore thymic function and potentially counteract age-related inflammation;^427,428^ (3) extracellular vesicles and exosomes derived from young, healthy serum might contribute to the recovery of thymic aging by enhancing FoxN1 expression, characterized by partial reversal of thymic involution and enhanced negative selection signals;^429^ and (4) furthermore, key cytokines and growth factors essential for maintaining TEC function, including mesenchymal-derived KGF, macrophages and T lymphocyte-derived IGF-1, and thymic stromal cell-derived bone morphogenetic protein-4 (BMP4), as well as critical TEC functions such as the production of cytokines (e.g., IL-7 and Kit ligands) and chemokines (e.g., SDF-1 and TECK), can improve thymic function in mice.^424,430–434^ Deficiency of the epithelium-specific microRNA-205 (miR-205) can lead to significant thymic involution.^435,436^

#### Bone marrow

Bone marrow (BM) can provide niches for plasma cells and memory T cells and is influenced by aging. Immunosenescence is widely acknowledged to play a role in the pathogenesis of AD.^437^ A previous study revealed that young bone marrow transplantation (BMT) can rejuvenate peripheral cells, alleviate aging-associated signaling pathways, restore altered intercellular communication in senescent PBMCs, and reduce the levels of SASP factors in the blood (Fig. 6).^438^ Pangrazzi et al. revealed that the expression of molecules responsible for sustaining immune memory in the human BM undergoes age-related alterations. On the basis of this insight, the application of antioxidants could mitigate inflammatory levels within the BM and promote the generation of adequate “appropriate” survival factors for memory cells. Consequently, such interventions may enhance the preservation of immune memory during aging.^439^ Most importantly, clarifying the concept of the hematopoietic niche, which refers to the spatial localization and unique microenvironment of HSCs and immune cells within the bone marrow, is essential. The critical role of HSCs in immunosenescence is clear. As the bone marrow serves as the site of the hematopoietic niche, targeting senescent niches within the bone marrow holds broad application prospects. For example, targeting HIF transcription factors (involved in ROS production pathways) has been shown to improve outcomes in patients with hematopoietic disorders by reconstructing the niche composition. Additionally, supplementation with β3-adrenergic receptor agonists can enhance sympathetic innervation in the bone marrow and promote hematopoietic function.

#### Peripheral immune organs

Although thymic regeneration represents a promising therapeutic strategy for addressing immunosenescence, emerging evidence suggests that age-associated fibrosis in lymph nodes may counteract the benefits conferred by thymic rejuvenation (Fig. 6).^440^ Consequently, lymph node fibrosis has emerged as a potential therapeutic target in the context of rejuvenating the aged immune system. Alternatively, the restoration of lymphatic and immune cell functions through the utilization of functional synthetic lymphoid organoids composed of stromal progenitor cells and decellularized extracellular matrix scaffolds represents a novel and innovative approach.^441^ In addition, as the largest secondary immune organ in the body, the spleen is closely associated with immune aging.^442^ Reports indicate that transplanting splenic cells from young mice can reduce aging and tissue damage in aged mice.^443^ Furthermore, systemic AAV vector-mediated LAV-BPIFB4 gene transfer has been shown to redirect circulating SnCs to the spleen and increase the activity of immune cells within the spleen, thereby effectively eliminating senescent immune cells.^444^ However, immune organs do not function in isolation, and targeting a single organ still has numerous limitations. Therefore, combining this approach with other strategies is essential to achieve long-lasting restoration of immune function.

### Nutritional and lifestyle interventions

A nutritionally optimized diet coupled with a balanced lifestyle exerts indispensable immunomodulatory effects through the potentiation of innate and adaptive immune responses, as well as the attenuation of chronic low-grade inflammation (inflammaging). These synergistic interventions constitute critical determinants in preventing age-related immunosenescence and mitigating the pathogenesis of various inflammation-associated disorders, including metabolic syndrome, cardiovascular diseases, and neurodegenerative conditions (Fig. 6).

Omega-3 polyunsaturated fatty acids (PUFAs), which are enriched in fatty fish and exhibit potent anti-inflammatory activities through multiple molecular mechanisms, are of particular interest.^445,446^ Extensive research has shown that omega-3 PUFAs exert pleiotropic regulatory effects on virtually all immune cell populations, including but not limited to T cells, B lymphocytes, macrophages, and neutrophils, thereby increasing their functional competence.^447–450^ Furthermore, clinical evidence has substantiated the adjunctive therapeutic efficacy of omega-3 PUFAs in the management of various pathological conditions.^445^ Of particular importance are the antioxidant compounds that play pivotal roles in cellular defense mechanisms, which are derived primarily from vegetable and fruit sources.^451,452^ These bioactive molecules play crucial roles in protecting cellular integrity against oxidative stress and modulating immune homeostasis. A notable example is vitamin C (ascorbic acid) and its stable derivative, L-ascorbic acid 2-phosphate, which significantly enhance the proliferative capacity and effector functions of γδ T cells, a unique subset of T lymphocytes that bridge innate and adaptive immunity.^453^ Emerging evidence indicates that exogenous stimuli, including fungal components, may drive and perpetuate immunosenescence.^454^ Strategic modulation of the gut mycobiota through dietary interventions, fecal microbiota transplantation, probiotic supplementation, and prebiotic administration has therapeutic potential in enhancing immunotherapy efficacy while mitigating immune-related adverse events.^455,456^

In addition to a healthy diet, regular moderate-intensity exercise has been demonstrated to enhance various aspects of immune function. A substantial body of literature confirms that consistent physical activity can reduce the levels of proinflammatory cytokines, including IL-6 and TNF-α, while increasing lymphocyte proliferation and NK cell activity. Furthermore, regular exercise helps maintain the mass and function of the thymus gland.^457–460^ Importantly, muscles act as modulators of the immune system. IL-15, produced by muscle, enhances the cytotoxicity of NK cells and increases cytokine secretion. Additionally, myokines, which are secreted by skeletal muscle, possess anti-inflammatory properties and contribute to the enhancement of immune responses.^196,461^ This evidence underscores the critical role of exercise in modulating immune responses, and the mechanisms underlying these benefits are thought to involve both systemic and cellular adaptations, including improved circulation of immune cells, reduced chronic low-grade inflammation, and enhanced stress resistance.^462^ Thus, integrating regular moderate exercise into lifestyle practices is a scientifically supported strategy for optimizing immune health. Chronic stress has been scientifically validated to accelerate immunosenescence, characterized by heightened systemic inflammation and a decline in immune efficacy.^463,464^ Interventions such as meditation, yoga, and controlled breathing techniques have demonstrated efficacy in attenuating stress responses and bolstering immune resilience.^463,465^ Concurrently, adherence to robust sleep hygiene protocols and the management of sleep disorders are imperative for sustaining immune homeostasis and overall physiological well-being.^466,467^ Empirical evidence indicates that smoking exacerbates immune aging, thereby increasing susceptibility to a spectrum of age-related pathologies.^468^ Smoking cessation has been associated with a reduction in the expression of proinflammatory markers and the restoration of immune cell functionality, including the normalization of T cells and NK cell dynamics.^469,470^ Furthermore, the timely administration of age-appropriate vaccines is crucial for counteracting the waning immune responsiveness observed in the elderly population.^471,472^ These multifaceted, evidence-based interventions underscore the pivotal role of lifestyle modifications in preserving immune integrity and ameliorating the deleterious effects of immunosenescence.

## Clinical applications

Various clinical trials have been conducted to investigate the aforementioned therapeutic strategies. For example, in the strategy of targeting immune organ rejuvenation, a phase II clinical trial demonstrated the potential of miR-205 mimics to restore thymopoiesis in settings of diminished or impaired T cell output. The underlying mechanism involves the upregulation of FOXN1 and FOXN1-regulated chemokines.^436^ Additionally, in the context of immune cell-targeted therapies, allogeneic hematopoietic stem cell transplantation (allo-HSCT) has emerged as a primary clinical modality for counteracting immunosenescence and treating associated conditions by targeting the restoration of HSC vitality and function (NCT06484049) (Table 2). Of particular interest is the continuous emergence of clinical trials utilizing de novo T cell generation to treat diseases. A phase I/IIa randomized, placebo-controlled, multicenter study reported that repeated administration of glycosylated recombinant human IL-7 (rhIL-7) in patients infected with human immunodeficiency virus-1 (HIV-1) may facilitate the achievement and maintenance of normal circulating CD4^+^ T cell counts. More importantly, this treatment promotes the expansion of rejuvenated T cell populations, such as naïve T cells (NCT0047732).^473^ Furthermore, rhIL-7 significantly expands the diversity of the circulating TCR repertoire. These findings suggest that rhIL-7 therapy can potentiate and broaden immune responses, particularly in individuals with limited naïve T cell populations and reduced TCR repertoire diversity, such as those experiencing advanced age, HIV infection, or iatrogenic (chemotherapy-induced) lymphodepletion.^474,475^ Consequently, rhIL-7 holds promise for maintaining T cell homeostasis, increasing T cell numbers, and enhancing their cytotoxic functions, not only in idiopathic CD4^+^ lymphopenia but also in the context of various cancer therapies (Table 2).^476^ Several clinical trials, including NCT00376935, NCT02356159, and NCT00593554, have aimed primarily to investigate the impact of recombinant human KGF (palifermin) on peripheral T cell reconstitution. However, palifermin may exacerbate thymic dysfunction following alemtuzumab treatment and should not be used to promote T cell recovery^392^. Importantly, palifermin is generally not utilized as a standalone therapeutic agent but rather in conjunction with other immune-enhancing therapies. Consequently, its potential to enhance immune recovery following T cell-depleting, total body irradiation-based hematopoietic stem cell transplantation is being explored when it is coadministered with leuprolide acetate (NCT01746849) (Table 2) or in combination with busulfan, melphalan, and fludarabine (NCT00629798). Allogeneic CAR-T cell therapy targeting B cell maturation antigens has demonstrated clinical feasibility in relapsed/refractory multiple myeloma.^477^ Similarly, other types of lymphocyte-B cells have been investigated to determine whether restoring B cell production in the bone marrow or reconstructing the peripheral B cell repertoire could enhance the immune responsiveness of elderly individuals to neoantigen challenges (NCT00863187) (Table 2). Furthermore, as highlighted in the previous section regarding therapeutic strategies, the critical relationship between immunosenescence and inflammation has prompted clinical trials to investigate targeting macrophages (NCT01045512), endothelial cells (NCT04537884), and neutrophils (NCT02441205) (Table 2) in an effort to mitigate immunosenescence and combat conditions such as congestive heart failure, neovascular age-related macular degeneration, and diabetes. However, owing to interindividual variability, specific genetic or epigenetic characteristics may influence therapeutic outcomes. Larger-scale clinical studies are needed to identify optimal treatment strategies that achieve the best health outcomes with minimal side effects.

**Table 2:** Clinical trials of targeted immune cells

Target cell	NCT number	Interventions	Conditions	Study title
Hematopoietic stem cell	NCT03940586	Drug: Letermovir oral granules Drug: Letermovir tablet Drug: Letermovir intravenous	Cytomegalovirus (CMV) infection	Letermovir Treatment in Pediatric Participants Following Allogeneic Hematopoietic Stem Cell Transplantation (HSCT)
	NCT06484049	Hematopoietic Stem Cell Transplantation	Hematopoietic stem cell transplantation	Prospective Observational Clinical Study on Changes in Cognitive Levels in Elderly Patients Before and After Hematopoietic Stem Cell Transplantation
	NCT06769568	Drug: reduced-dose conditioning regimen containing TBI in hematopoietic stem cell transplantation treating elderly patients with aplastic anemia	Aplastic anemia	Reduced-dose Conditioning Regimen Containing TBI in HSCT Treating Elderly Patients With Aplastic Anemia
	NCT06399107	Genetic: Drug Product is administered by IV infusion following myeloablative conditioning with busulfan	Sickle cell disease	Investigation Into the Use of BAH243 Lentiviral Vector for Gene Therapy in Treating Sickle Cell Disease (BAH243)
T cell	NCT05037669	Biological: PACE CART19	Acute lymphoblastic leukemia, Chronic lymphocytic leukemia	Programmed Allogeneic CRISPR-edited T Cells Engineered to Express Anti-CD19 Chimeric Antigen Receptor (PACE CART19) in Patients With Relapsed Or Refractory CD19^+^ Leukemia and Lymphoma
	NCT05979363	Biological: anti-BCMA CAR-T Drug: VRD-based regimen	Plasma cell leukemia	A Study of Bortezomib, Lenalidomide and Dexamethasone (VRd) Followed by BCMA CAR-T Therapy in Transplant-Ineligible Patients With Primary Plasma Cell Leukemia
T cell	NCT04093596	Genetic: ALLO-715 Biological: ALLO-647 Drug: Fludarabine Drug: Cyclophosphamide Drug: Nirogacestat	Relapsed/Refractory Multiple Myeloma	Safety and Efficacy of ALLO-715 BCMA Allogenic CAR T Cells in in Adults With Relapsed or Refractory Multiple Myeloma (UNIVERSAL) (UNIVERSAL)
	NCT00477321	Drug: CYT 107	HIV Infections, Lymphopenia	Safety Study of IL-7 in HIV-infected Patients (Inspire)
	NCT01190111	Biological: Interleukin-7	HIV	Study on Interleukin-7 (CYT107) in HIV Patients (Inspire 2)
	NCT00684008	Drug: CYT107 – Recombinant glycosylated human interleukin 7. Drug: rhIL-7 (CYT107)	AML, CML, MDS	Safety Study of IL-7 in Recipients of a Hemopoietic Stem Cell Transplant Peripheral Blood Stem Cell Transplant
	NCT01233921	Biological: palifermin Other: flow cytometry Other: laboratory biomarker analysis Other: pharmacological study	Accelerated Phase Chronic Myelogenous Leukemia, Adult Acute Lymphoblastic Leukemia in Remission	Palifermin in Preventing Chronic Graft-Versus-Host Disease in Patients Who Have Undergone Donor Stem Cell Transplant for Hematologic Cancer
	NCT01746849	Biological: Palifermin Biological: Lupron Procedure: peripheral blood stem cell transplantation Radiation: Total-Body Irradiation (TBI) Drug: Thiotepa Drug: Cyclophosphamide Drug: Degarelix	Non-Hodgkin’s lymphoma, Myelodysplastic syndrome, Multiple myeloma, Leukemia	Palifermin With Leuprolide Acetate for the Promotion of Immune Recovery Following Total Body Irradiation Based T-Cell Depleted Allogeneic Hematopoietic Stem Cell Transplantation
B cell	NCT00863187	Biological: rituximab	Lymphoma	Assessing Antibody Responsiveness to Hepatitis B Vaccine in Aged Lymphoma Patients Undergoing Treatment With Rituximab
	NCT04146285	Drug: BAT4406F	Neuromyelitis optica spectrum disorders	A Phase I Clinical Trial of BAT4406F Injection in Patients With Neuromyelitis Optica Spectrum Disorders
NK cell	NCT06161545	Drug: N-803 Drug: pembrolizumab Biological: PD-L1 t-haNK cells	Stage II squamous cell carcinoma of the head and neck, stage III Squamous Cell Carcinoma of the Head and Neck, Stage IV Squamous Cell Carcinoma of the Head and Neck	embrolizumab + N-803 Alone or in Combination With PD-L1 t-haNK Cells for Resectable Head and Neck Squamous Cell Carcinoma
	NCT04646980	Dietary Supplement: Biobran/MGN-3 Other: Placebo	Influenza-like Illness	Biobran/ MGN-3 Increases Innate Resistance and Reduces the Incidence of Influenza-like Illnesses
Macrophage	NCT03487679	Other: Fasting	Fasting	Effects of Prolonged Fasting on Microbiome and HDL
	NCT01686568	Drug: Omega-3 Drug: placebo	Insulin Resistance	Omega-3 Fatty Acids and Insulin Sensitivity
	NCT00473876	Drug: Metformin Drug: Matched Placebo (Capsules)	Congestive heart failure, Insulin resistance	Metformin in Insulin Resistant Left Ventricular (LV) Dysfunction (TAYSIDE Trial) (TAYSIDE)
	NCT01045512	Drug: fluvastatin	Aging, Inflammation	The Role of the “Inflammatory/ Pathogen Burden” for Cardiac Ageing (AntiCardAgeing)
Endothelial cell	NCT04537884	Drug: UBX1325	Diabetic macular edema, Neovascular age-related macular degeneration	Safety and Tolerability Study of UBX1325 in Patients With Diabetic Macular Edema or Neovascular Age-Related Macular Degeneration
	NCT02494141	Drug: Curcumin Other: Placebo	Polycystic kidney, Autosomal dominant	Curcumin Therapy to Treat Vascular Dysfunction in Children and Young Adults With ADPKD
Neutrophil	NCT02441205	Behavioral: High Intensity Interval training	Aging, Disease	Interval Training, Inflammation and Immune Function
Dendritic cell	NCT03026244	Dietary Supplement: Milk protein, prebiotics, vitamin D Dietary Supplement: Placebo product	Immunosenescence, Inflammation	Effect of Milk Protein and Prebiotics in Combination With Vitamin D on Innate Immunity in Elderly People
Eosinophil	NCT02305940	Drug: Doxycycline Drug: Placebo	Chronic obstructive pulmonary disease (COPD)	Effects of Long Term Antibiotic Therapy on Exacerbation Rate in Stable COPD Patients
Basophil	NCT05346302	Drug: Pneumovax 23 Drug: Typhim VI Other: Saline	Inflammatory Response	Persistent Readiness Through Early Prediction Immunization Study (PREP DOD)

*HIV* human immunodeficiency virus, *AML* acute myeloid leukemia, *CML* chronic myelogenous leukemia, *MDS* myelodysplastic syndrome, *PD-L1* programmed cell death ligand 1, *HDL* high-density lipoprotein. Source: https://clinicaltrials.gov/

Even more intriguing is that advancements in artificial intelligence (AI) have the potential to significantly propel the field of personalized medicine forward. By leveraging vast databases, AI can analyze large-scale genomic and epigenomic data to identify patients who are most likely to benefit from specific rejuvenation therapies.^478^ This capability is based on age-related changes in the immune system and alterations in signaling pathways to predict treatment outcomes, design clinical trials, and tailor personalized therapeutic strategies.^478,479^ For example, a recently reported integrated bioinformatics analysis utilized machine learning to extract a predefined immunosenescence index from aggregated gene expression data, which can predict treatment outcomes and drug sensitivity in melanoma patients.^480^ Furthermore, the predictive modeling capabilities of AI can help biologists gain deeper insights into the mechanisms and biological implications of immune aging. Machine learning algorithms can analyze high-dimensional datasets (genomic, transcriptomic, and proteomic) to identify the most relevant biomarkers and potential therapeutic targets, thereby reducing time costs and enhancing treatment efficiency.^481,482^ Similarly, using AI to track the patient’s physical changes in real time throughout the whole process is conducive to medical staff’s understanding of the patient’s treatment effect and recovery ability, thus providing convenience for medical staff to update the treatment plan.^483^

## Conclusions and future directions

In summary, this review delineates the multifactorial determinants underlying the pathogenesis of immunosenescence and its contributory role in diverse age-related pathologies. The dysregulation of immunosenescence-associated signaling pathways and the unique phenotypic alterations observed in senescent immune cells within these regulatory networks were summarized. Emphasis is placed on how to use targeted therapies, immunomodulatory strategies at the cellular and organ levels, or nutritional and lifestyle interventions to fight immunosenescence. Clinical advances in the development of immunosenescence-targeted treatment methods are also discussed.

Given the inextricable link between immune system dysregulation and organismal aging, future research on preventing or mitigating immunosenescence may prioritize the integrated application of the therapeutic strategies discussed herein. The therapeutic strategy mentioned in the text involves targeting immune organs to counteract immunosenescence by restoring thymic vitality, rejuvenating bone marrow niches, and improving the function of peripheral immune organs, ultimately enhancing the generation and functionality of immune cells. Targeting various immune cells aims to restore their cellular functions, with T cells being the most critical. The senescence of T cells can be reversed through IL-7^473^ and KGF^391,392^ therapies, thereby ameliorating associated pathological conditions. Inhibiting NF-κB reverses SASP and delays aging-related pathologies via IKK/NF-κB inhibitors (e.g., 8K-NBD) or phytochemicals such as curcumin and resveratrol.^10,278,279,288^ MTORC1 inhibition (e.g., rapamycin, RapaLink-1) extends lifespan by enhancing autophagy and metabolic regulation, with clinical trials targeting immunosenescence.^295–297,302^ Mitigating immunosenescence can be achieved through dietary optimization, regular moderate exercise, stress management, sufficient sleep, smoking cessation, and timely vaccination. Crucially, synergistic interplay between these modalities, such as combining senolytics with epigenetic reprogramming or mTOR inhibition, should be rigorously explored to increase efficacy while minimizing unintended adverse effects. A central conundrum in immunosenescence research lies in navigating the dual risks of therapeutic interventions: suppressing age-associated inflammation (“inflammaging”) may inadvertently exacerbate infection susceptibility or compromise basal immune homeostasis, whereas overactivating inflammatory pathways to restore immune function risks precipitates autoimmune pathologies or acute inflammatory sequelae. Achieving this delicate equilibrium demands precision in intervention dosing. Notably, the integration of personalized medicine frameworks and AI into immune aging management is a strategic but challenging frontier. As this field evolves, interdisciplinary collaborations-spanning immunology and computational biology—will be integral to translating these insights into therapies that improve immune resilience and healthy longevity.

In the past few decades, our understanding of immunosenescence has increased, and various treatment methods, such as immunological intervention measures and pathway-targeted drugs, have emerged. However, despite the existence of many treatment methods, the biological changes and underlying mechanisms behind immunosenescence remain unclear. The effects of a single treatment method are not ideal, and various side effects faced in clinical translation hinder the treatment of immunosenescence. In addition, aging has always been regarded as a natural process, and the use of various means to impede this biological process may present many doubts. For example, the genetic changes introduced by gene editing can be passed on to future generations through germline modification, which may lead to unforeseeable genetic complications or ecological impacts on the human gene pool. This poses considerable ethical challenges in both the health and social fields. Moreover, although personalized medicine provides one-on-one treatment for patients, which further improves the treatment plan for patients and is conducive to their further recovery and improvement in their quality of life, the associated cost may be high for ordinary people. As a result, these personalized medical treatments may become exclusive to wealthy individuals or countries, thus deepening the global health gap. Artificial intelligence and machine learning have broad prospects in the field of aging treatment and the importance of further research and development. However, the data security issues they cause are the greatest concern for people. How to balance the relationship between the security of patients’ private data and the transparent management of artificial intelligence is the key to achieving a win‒win situation through the cooperation of various disciplines.
